# Fluvial Depositional Systems of the African Humid Period: An Analog for an Early, Wet Mars in the Eastern Sahara

**DOI:** 10.1029/2021JE007087

**Published:** 2022-05-13

**Authors:** A. S. Zaki, J. M. Davis, K. S. Edgett, R. Giegengack, M. Roige, S. Conway, M. Schuster, S. Gupta, F. Salese, K. S. Sangwan, A. G. Fairén, C. M. Hughes, C. F. Pain, S. Castelltort

**Affiliations:** ^1^ Department of Earth Sciences University of Geneva Geneva Switzerland; ^2^ Department of Earth Sciences Natural History Museum London UK; ^3^ Malin Space Science Systems, Inc. San Diego CA USA; ^4^ Department of Earth & Environmental Science University of Pennsylvania Philadelphia PA USA; ^5^ Department de Geologia Universitat Autònoma de Barcelona Barcelona Spain; ^6^ CNRS UMR 6112 Laboratoire de Planétologie et Géodynamique, Université de Nantes Nantes France; ^7^ Université de Strasbourg CNRS Institut Terre et Environnement de Strasbourg Strasbourg France; ^8^ Department of Earth Sciences and Engineering Imperial College London London UK; ^9^ Centro de Astrobiología (CSIC‐INTA), Torrejón de Ardoz Madrid Spain; ^10^ International Research School of Planetary Sciences (IRSPS) Università d’Annunzio Pescara Italy; ^11^ Department of Astronomy Cornell University Ithaca NY USA; ^12^ Department of Geosciences University of Arkansas Fayetteville AR USA; ^13^ MED_Soil, Departamento de Cristlografía, Mineralogía y Quimica Agrícola Universidad de Sevilla Sevilla Spain

**Keywords:** fluvial ridges, inverted channels, relief inversion, african humid period, deltas, earth analogs

## Abstract

A widely hypothesized but complex transition from widespread fluvial activity to predominantly aeolian processes is inferred on Mars based on remote sensing data observations of ancient landforms. However, the lack of analysis of in situ martian fluvial deposits hinders our understanding of the flow regime nature and sustainability of the martian fluvial activity and the hunt for ancient life. Studying analogs from arid zones on Earth is fundamental to quantitatively understanding geomorphic processes and climate drivers that might have dominated during early Mars. Here we investigate the formation and preservation of fluvial depositional systems in the eastern Sahara, where the largest arid region on Earth hosts important repositories of past climatic changes. The fluvial systems are composed of well‐preserved single‐thread sinuous to branching ridges and fan‐shaped deposits interpreted as deltas. The systems' configuration and sedimentary content suggest that ephemeral rivers carved these landforms by sequential intermittent episodes of erosion and deposition active for 10–100s years over ∼10,000 years during the late Quaternary. Subsequently, these landforms were sculpted by a marginal role of rainfall and aeolian processes with minimum erosion rates of 1.1 ± 0.2 mm/yr, supplying ∼96 ± 24 × 10^10^ m^3^ of disaggregated sediment to adjacent aeolian dunes. Our results imply that similar martian fluvial systems preserving single‐thread, short distance source‐to‐sink courses may have formed due to transient drainage networks active over short durations. Altogether, this study adds to the growing recognition of the complexity of interpreting climate history from orbital images of landforms.

## Key Points

- Ancient depositional rivers in southern Egypt record ephemeral fluvial systems formed due to intense rainfall over ∼10 ka
- These fluvial systems suggest tens to hundreds of years of river activity
- Similar martian systems imply that early Mars's surface was punctuated by local and transient drainage systems fed over short durations

## 1 Introduction

Mars is currently hyperarid, but considerable geological evidence suggests that the martian surface was at least episodically warm during the late Noachian and early Hesperian ca. 3–4 Ga (e.g., Malin & Edgett, 2003; Fassett & Head, 2008; Davis et al., 2016; Salese et al., 2019; Wordsworth, 2016; Kite, 2018; Dickson et al., 2020). This warming epoch might have triggered both ice melting and hydrological cycle activation, forming valley networks, paleolakes, and fan‐shaped deposits (e.g., Di Achille & Hynek, 2010; Goudge et al., 2012, 2018; Gulick, 2001; Kite, 2018; Mangold et al., 2004; Wordsworth, 2016). Following these periods, Mars transitioned from wet‐to‐dry conditions, likely driven by reductions in atmospheric pressure (Lammer et al., 2013), although the precise nature of this transition is unclear (Kite et al., 2019). Since the Hesperian, the martian landscape has been exposed to prolonged aeolian erosion, revealing much of the planet's ancient geologic record. Former stream deposits (at all scales from rill to creek to river) are now exposed as ridges at numerous locations across the martian surface in a variety of settings (e.g., Burr et al., 2010; Cardenas et al., 2017; Davis et al., 2019; Di Pietro et al., 2018; Dickson et al., 2020; Williams, 2007; Zaki, Pain, et al., 2021). Some of these ridges have been used to reconstruct the paleohydrology and paleoclimate of early Mars (Burr et al., 2010; Davis et al., 2016; Kite et al., 2019; Hayden, Lamb, & Carney, 2021; Hayden, Lamb, & McElroy 2021).

**Figure 1:**
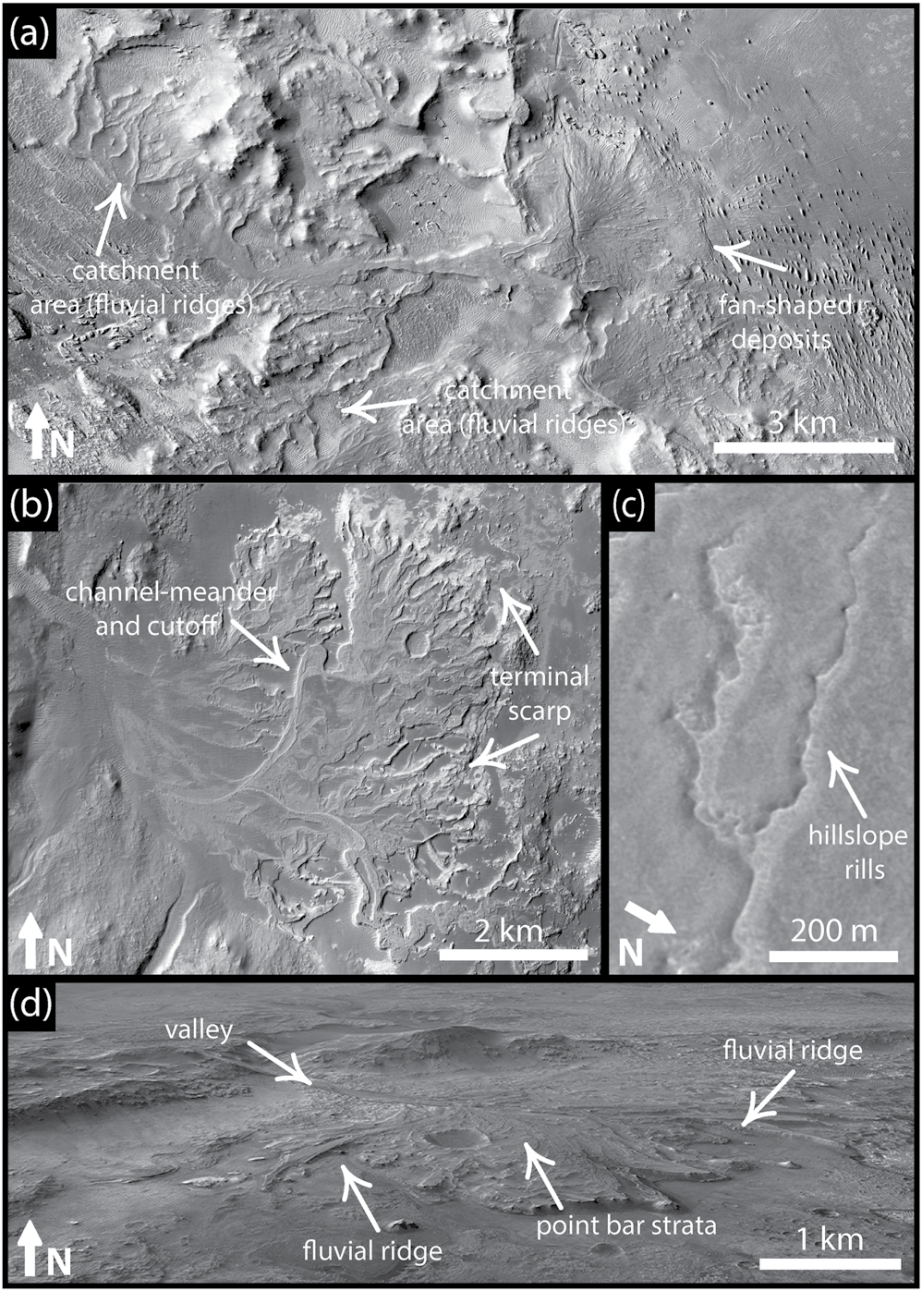
Topographically inverted landscape features: (a) A short‐distance source‐to‐sink paleo‐river, now expressed as a set of ridges and a fan in the modern landscape, in the western Medusae Fossae Formation region of Mars. The entire source to sink fluvial system stands as ridges in the modern landscape (CTX image P21_009109_1797_XN_00S213W; 0°15'52.24"S; 146°25'26.07"E). (b) Deltaic deposit in Eberswalde crater that preserves many topographically inverted paleochannels (CTX image P01_001534_1559_XI_24S033W; 23°49'9.76"S; 33°39'45.97"W). (c) Fluvial ridges along on the plateau west of Juventae Chasma (CTX image G06_020602_1757_XN_04S063W 4°14'35.67"S; 63°14'34.09"W). (d) Perspective view of the Jezero crater “western delta” showing ridges interpreted by (Goudge et al., 2018) to be inverted channels (Credit: NASA/JPL/Seán Doran; 18°33'19.90"N; 77°23'36.79"E).

Such ridges on Earth result from a cyclic process in which ancient rivers were filled with resistant sediment, including volcanic products, large clasts, and/or cemented clasts, and were subsequently topographically inverted by differential erosion to stand as ridges in the modern landscape (e.g., Maizels, 1983, 1987, 1990; Williams et al., 2007, 2009, 2021; Foix et al., 2012; Madof et al., 2019; Pain & Ollier, 1995; Zaki, Pain, et al., 2021). In some cases, such ridges appear in the landscape after deep burial, followed by exhumation and net landscape lowering by differential erosion (e.g., Balme et al., 2020; Cardenas et al., 2020; Davis et al., 2019; Hayden et al., 2019; Ielpi & Ghinassi, 2014; Zaki, Pain, et al., 2021). Ridges are observed in different forms, including flat‐topped, elongated sinuous ridges, fan‐shaped deposits (deltas and fluvial fans), meanders, and point bars (Figure 1; Davis et al., 2016; Cardenas et al., 2020; Hayden et al., 2019; Hayden, Lamb, & Carney, 2021; Hayden, Lamb, & McElroy, 2021; Dickson et al., 2020; Zaki, Pain, et al., 2021). The investigation of these landforms on Mars is now only possible through remotely sensed data from orbiters (although this might soon change, at least in Jezero crater, when the Perseverance rover arrives at the deltaic landform in Figure 1d; Mangold et al., 2021), hindering our ability to measure their exact geometries, understand their development, how much time they record, and the mechanisms of their preservation (e.g., sediment cementation) during the shift from wet to arid environments. Although fluvial ridges on Earth have been widely investigated (e.g., Pain & Ollier, 1995; Williams et al., 2007, 2009, 2021, Clarke et al., 2020; Hayden et al., 2019; Hayden, Lamb, & Carney, 2021; Hayden, Lamb, & McElroy, 2021; Zaki et al., 2018; Zaki, Pain, et al., 2021, further studies of similar landforms in dry climates on Earth help provide uniformitarian analogs of the physical principles of landform evolution processes, including streamflow mechanisms, duration of fluvial activity, patterns of fluvial deposition, processes of relief inversion, and the nature of the transition from wet to arid climates. Constraining such information can refine our understanding of martian landforms with similar planforms and sedimentary structures by providing new insights into the paleoenvironmental conditions associated with their development. Such information may also improve our understanding of habitability and potential biosignature on early Mars.

**Figure 2:**
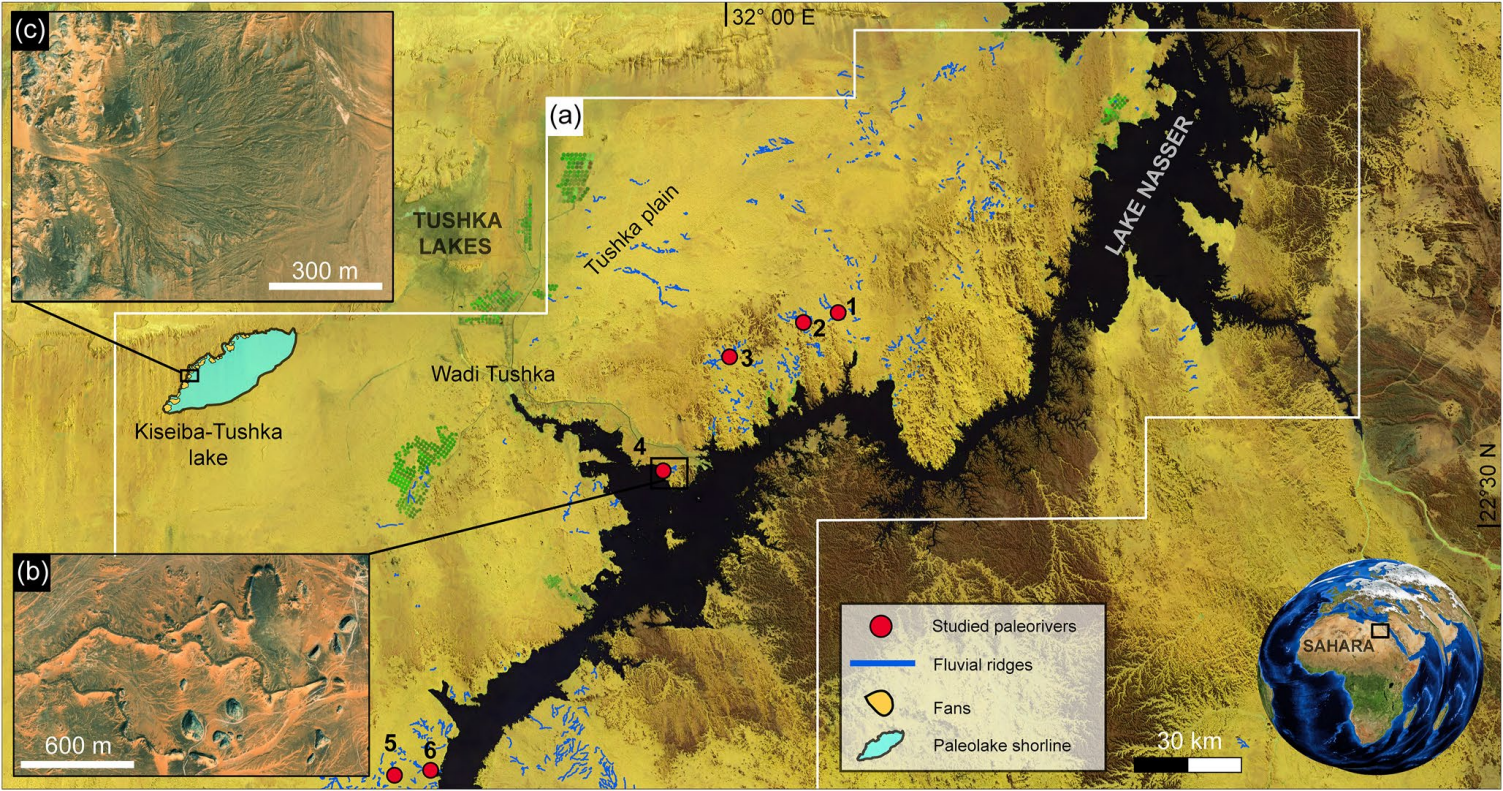
(a) Locations of fluvial ridges and paleodeltaic landforms in the eastern Sahara mosaic of Landsat 8 images acquired in 2018 overlaid on a shaded relief map produced from Shuttle Radar Topography Mission (SRTM). The paleorivers discussed in this paper are; (1) Gabal Hamam I, (2) Gabal Hamam II, (3) Gabal Masmas, (4) Gabal El‐Sadd, (5) Arqin I, and (6) Arqin II. (b) Example view of a dendritic fluvial ridge system, located at 22°37'36.56"N; 31°47'33.18"E (Portion of Esri World Imagery product created in 2009). (c) Example of fan‐shaped landform and inverted paleochannels (paleodelta) at the western shore of dry lake Kiseiba‐Tushka at 22°48'31.49"N; 30°46'56.48"E (Portion Esri World Imagery product created in 2009).

**Figure 3:**
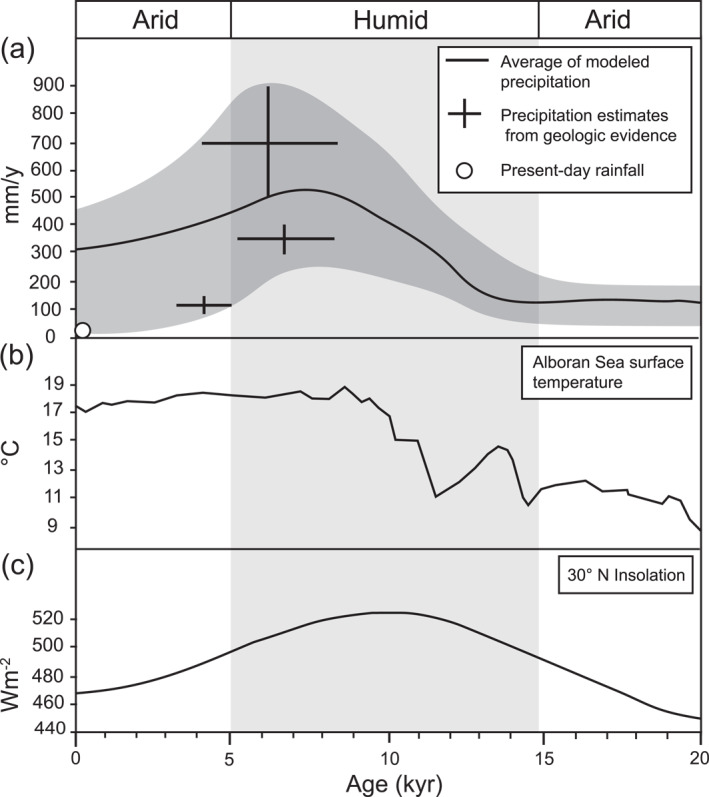
Environmental proxies from the eastern Sahara show an increase in insolation that promoted higher temperature, leading to wetter conditions between 14.8 and 5.5 ka BP (the AHP). (a) Mean annual precipitation during the AHP as reported from Selima Oasis (Haynes et al., 1989), West Nubia Paleolake Basin (Hoelzmann et al., 2000), Bir Atrun and Oyo (Ritchie & Haynes, 1987), and Lake Yoa, Tibesti and Coastal Libya (Blanchet et al., 2021), and Lake Ohrid (Wagner et al., 2019). (b) Sea surface temperature reconstructing from the Alboran Sea (Martrat et al., 2004). (c) Summer insolation at 30°N (Berger & Loutre, 1991). Increasing the insolation during the AHP was driven by orbital changes.

The Sahara Desert, the largest dry region on Earth, contains superb repositories of past climatic and environmental changes from wet to arid conditions recorded by multiple geologic proxies. The record of the fluvial activity has been preserved as fossilized fluvial landforms due to the hyper‐arid conditions. A snapshot of this record is well‐preserved in southern Egypt, where ancient riverbeds and fans survive as inverted topography in the modern landscape. These landforms occur over an ∼38,000 km^2^ region on the western and eastern sides of Lake Nasser (Figure 2; Zaki & Giegengack, 2016; Giegengack & Zaki, 2017). These landforms are well‐preserved in inverted topography, taking different forms, including fine‐branched ridges, single‐thread ridges, and sedimentary fans (bearing inverted channel forms). Clastic sediments preserved in these landforms are well‐cemented (e.g., Giegengack & Zaki, 2017). After cementation, the sediments were left standing as ridges by a regional lowering of the desert surface due to aeolian and fluvial erosion during a period of climatic oscillation (Giegengack & Zaki, 2017). The discovery of Acheulian artifacts and pottery shards within the fluvial deposits stacked in the ridges established their relative age from middle Pleistocene to Holocene (Giegengack & Zaki, 2017). Furthermore, Zaki, King, et al. (2021) recently dated these paleo‐rivers using OSL and ^14^C; their results indicate that they were formed by several depositional events ranging from 53 ka to 1 ka. The ages indicate intense fluvial activity during the early (∼13,000 BP) to middle (∼5,200 BP) Holocene driven by heavy rainfall events (Zaki, King, et al., 2021). The intensity of rainfall events was briefly calculated using grain size and paleo‐channel geometry to obtain the discharge, then dividing the discharge by drainage area leads to minimum precipitation rates (Zaki, King, et al., 2021). This intense episode of humid conditions has been linked to the African Humid Period ‐ AHP (ca. 14.8 – ca. 5.5 ka BP; deMenocal et al., 2000; Hoelzmann et al., 2000; Nicoll, 2004; Schuster et al., 2005; Palchan & Torfstien, 2019). The AHP is an episode of increased insolation due to orbital changes, leading to a rise in temperatures by 7.5°C and precipitation by 3–4 times more before the AHP, turning the Sahara into a savannah‐like environment (Figure 3; deMenocal et al., 2000; Martrat et al., 2004; Kuper & Kropelin, 2006, Hoelzmann et al., 2000). After the termination of the AHP, the wet conditions abruptly ceased; this region today receives <2 mm per year of rainfall; thus, aeolian processes prevail (Hamdan et al., 2015; Nicoll, 2004).

In this contribution, we address the fundamental issues outlined above by investigating a diverse array of fluvial landform remnants, which represent a natural laboratory to quantitively understand the duration and the nature of the flow regime associated with similar martian features. We compiled remotely sensed data, field observations and measurements, petrographic observations, empirical relations, and published chronological data to reconstruct these landforms' formation, preservation, and evolution through time. These reconstructions provide new insights into the martian fluvial history, which may have undergone a similar drying to the Quaternary Sahara.

## 2 Regional Setting

The southeastern part of the Western Desert of Egypt (the eastern Sahara) covers an area of ∼50,000 km^2^. The landscape includes vast plains, cuestas, small basins, sporadically distributed hills, and aeolian dunes. The region can be broken into three physiographic units (Hamdan et al., 2016), the Sinn el‐Kiddab Plateau, the Tushka Plain, and Lake Nasser. The Sinn el‐Kiddab Plateau is underlain by horizontal limestone and shale units that were deposited during late Cretaceous to early Eocene time (Figure S1 in Supporting Information S1). This plateau now stands at an elevation of 300–400 m and overlooks the western side of the Nile. (Equation 2) The Tushka Plain lies south of the Sinn el‐Kaddab Plateau, borders Lake Nasser to the east, and extends to Sudan in the south. This ∼13,150 km^2^ plain is underlain by the Nubia Formation, primarily quartz‐rich sandstones of mid‐to‐late Cretaceous age; this terrain is partially overlain by a veneer of ephemeral playa lakes and associated sedimentary features that have characterized this landscape during wetter conditions during the late Quaternary time (e.g., Maxwell et al., 2010; Nicoll, 1998, 2004; Wendorf & Schild, 1980). Lake Nasser covers ∼5,200 km^2^ and has the capacity to hold ∼165 km^3^ of water.

This paper reconstructs the configuration of the former river systems that drained the Tushka Plain. Sediment deposited in these rivers now stands as ridges in the landscape (e.g., Giegengack & Zaki, 2017). Most of these paleorivers were developed on consolidated sedimentary quartzite, the “Nubia Formation”, deposited during the late Cretaceous time (Said & Issawi, 1964; Zaki & Giegengack, 2016). The Nubia Formation consists of detrital quartzite, derived mainly from the African Shield (Giegengack & Zaki, 2017). The grain size of clasts in this formation is highly variable, ranging from cobbles in the coarse basal conglomerates to silt and clay in the finer mudstone layers (Giegengack & Zaki, 2017). The Nubia Formation unconformably overlies an igneous and metamorphic complex (Giegengack & Zaki, 2017). The Nubia formation in the region was buried, lithified, then exhumed. Tewksbury et al. (2017) estimated the burial depth of the Nubia Formation at this region to fall in the range of ∼400–∼800 m. Then, it has been exhumed by tectonic uplift, coupled with a regional differential erosion (Tewksbury et al., 2017).

We also used remotely sensed data for a preliminary investigation of fan‐shaped deposits that accumulated at the margins of a ∼180 km^2^ paleolake basin and are now preserved, in part, as inverted topography. This paleolake basin, Kiseiba‐Tushka, occurs ∼40 km west of the fluvial‐ridge sites. Presently (2022), Kiseiba‐Tushka is filled with water diverted from Lake Nasser via the Wadi Tushka as a result of recent Nile flooding. The Kiseiba‐Tushka paleolake basin is one of a suite of shallow closed depressions, primarily wind‐deflated, in southern Egypt and northern Sudan that held shallow playa lakes during a wet episode that lasted from early to mid‐Holocene time (Nicoll, 1998). The paleolake basin hosts well‐preserved catchment areas, fans, and overlying aeolian sand dunes, and thus it records a hydroclimatic transition from wet to arid conditions.

The southeastern part of the Western Desert region is hyperarid, presently receiving <2 mm/yr of rainfall. However, regional climatic records document extreme events of heavy rainfall that occur sporadically every 10–30 years over the Egyptian Sahara, with rainfall as much as tens of millimeters in one day (Embabi, 2018). The wind frequently blows from the north‐northwest across northern Sudan and southern Egypt at speeds ranging from 4 to 20 km/hr (Oliver, 1965).

## 3 Materials and Methods

Unhindered by vegetation because of the hyperarid setting, we used a set of remotely sensed data (particularly satellite images) to identify and measure the geomorphic features of the fluvial ridges and the deltaic landform in the study region. These data include very high spatial resolution (∼50 cm/pixel) visible wavelength images obtained from the Pléiades satellite constellation. We also examined a combination of freely available satellite and aerial images presented at 2.5–15 m/pixel from Esri World Imagery and Bing Imagery, multispectral Landsat 8 scenes satellite at a scale of ∼30 m/pixel. Declassified CORONA satellite images were used to characterize geomorphic features now submerged beneath Lake Nasser. We also employed digital elevation data from the Japanese Advanced Land Observation Satellite (ALOS) with a spatial resolution of ∼28 m and a vertical resolution of 5 m to extract the drainage areas, river profiles, and slopes.

Zaki, King, et al. (2021) focused on obtaining absolute ages using OSL and ^14^C, and reconstructing precipitation rates involved in forming the upstream paleochannels of these fluvial systems based on grain size. However, investigating the morphology and the drainage areas from the source to sink systems, the internal architecture of the whole ridge, reconstructing discharge using channel width, and calculating the inversion rates remain unconstrained. Such parameters are essential for martian ridges, as we do not see the paleochannel geometries and grain size distribution from orbiter data; we instead see ridges.

**Figure 4:**
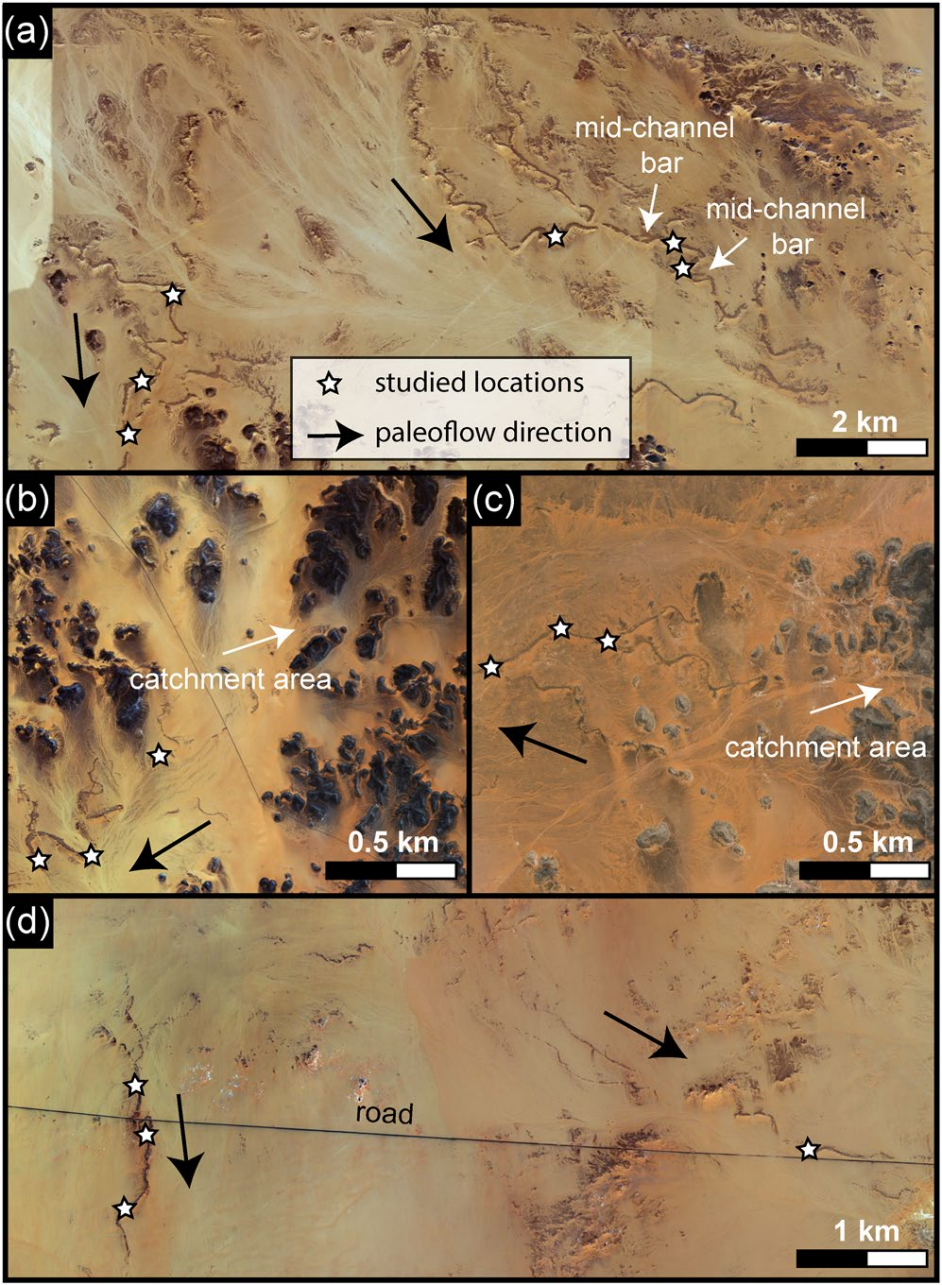
Satellite images of the six fluvial ridge sites showing the 16 sites we investigated along the paleo‐rivers (Credit: Bing Imagery). The white stars represent the examined sites. (a) Gabal Hamam I and II sites, (b) Gabal Masmas, and (c) Gabal El‐Sadd, and (d) Arqin I and II sites. The images also show that the ridges bear the dendritic pattern with their flow direction. The catchment areas are well‐expressed in portions B and C, where there are the Gabal Masmas and the Gabal El‐Sadd paleorivers.

Our fieldwork included measurement of fluvial ridge heights, widths, and thicknesses of stratigraphic sections deposited in the paleorivers at 16 locations along the six ridge systems (Figure 4; Table S1 in Supporting Information S1). We also observed and documented the sedimentary structures and facies within the ridge‐sediment sequences, including the storeys stacked in the ridges, imbrication, and bedding. In order to measure the grain size of these paleo‐rivers, we followed a method established by Wolman (1954) to determine the distribution of materials in coarse‐bedded streams. At each location, grain size data were obtained by measuring the b‐axes of 101–124 clasts of >2 mm size that were randomly picked out of a 1 m^2^ area following Wolman (1954). That is followed by determining the median of the grain size (*D*_*50*_) at each location based on multiple measurements.

We used satellite images to measure the lengths of the longest mainstream (*L*) of each of the fluvial ridge systems from the source to the sink to reconstruct the paleo‐drainage area (*A*) of each paleoriver via applying an empirical relationship developed by (Hack, 1957) (Equation 1) to estimate the area of the river basin. However, Som et al. (2009) suggested that the Hack's exponent (0.5–0.6) might lead to uncertainty in drainage area calculations in the case of ephemeral rivers. We, therefore, compared our drainage area estimates to modern rivers surviving under different fluvial settings, including sustained and flashy depositional systems (e.g, Patton & Baker, 1976; Church & Rood, 1983; Trampush et al., 2014). The flashy depositional environments are typically caused by intense rainfall over short‐duration events on a fast‐responding landscape (Patton & Baker, 1976). This contradicts the sustained depositional settings that required large drainage areas under enhanced rainfall.

(1) $$L=1.4A^{0.6}$$

Zaki, King, et al. (2021) used paleochannel geometries, particularly channel depth and width and grain size measurements, to calculate the paleoslope, paleovelocity, and paleodischarge of the ridges in southern Egypt. However, this approach is not feasible on Mars since most of the data about fluvial ridges is obtained from orbiter data. To reconstruct the discharge, we used a width‐based equation (Equation 2) developed by (Eaton, 2013), applied and showed high accurate estimates on Earth by Jacobsen and Burr (2018), and scaled to martian gravity by Kite et al. (2015) to estimate the discharge and the difference between using the two estimates. This might be helpful for a better understanding of paleohydaulics of similar features on Mars. Recent studies show that ridge width does not reflect the original channel width, and it should be larger in the case of the presence of ridges preserving channel belts (Hayden et al., 2019; Hayden, Lamb, & Carney, 2021). The rivers discussed in this paper, however, record channel fill with an absence of vertical aggradation and lateral migration. The ridge width, therefore, is nearly equal to the channel width, particularly in this type of stratigraphic architecture (e.g., Hayden et al., 2019). However, the ridges investigated in this study record channel fill that closely preserve the original channel shape (Hayden et al., 2019).

(2) $$Q=0.10W^{1.866}$$

Our petrographic interpretations were based on a study of 31 thin‐sections. (2.3*4 cm) under a petrographic microscope for a better understanding of the paleoenvironmental conditions associated with the source rocks. The environmental information that can be obtained from thin‐sections are the nature of deposition, cementation processes, and burial conditions. The environmental conditions can be inferred from the type of mineral, the shape and the size of the grain, and the features that were overprinted on the rocks due to differential processes throughout time (Mackenzie et al., 2017).

We calculated migration rates for meanders in the Kiseiba‐Tushka paleolake basin deltaic landform using an empirical relation developed by Lapôtre and Ielpi (2020) through a global compilation of channel migration rates of the channel width (*w*) of both vegetated and unvegetated rivers. We applied this approach to the equation of vegetated rivers (Equation 3), assuming—based on the pollen record in the eastern Sahara during the AHP (Haynes et al., 1989; Ritchie & Haynes, 1987)—that the studied rivers were formed under vegetated environments. To get the duration we multiplied the migration distance by the migration rate to constrain how many years of bankful flows were required to form the meander belt.

(3) $$Mr_{Ev}=\left(0.023\pm 0.001\right)w^{0.85}$$

Differential erosion has lowered the desert surface to produce these ridges. We can estimate the minimum erosion rate (*η*) at each ridge by dividing the ridge height (*H*) by the age (*T*) of the youngest fluvial sediment in that ridge (Wang et al., 2015) (Equation 4). The ages of these ridges were measured using ^14^C and optically stimulated luminescence (OSL) (Zaki, King, et al., 2021), but we also used the ages derived from other proxies, including lake sediments and levels, pollen, and dust fluxes (Hoelzmann et al., 2000; Palchan & Torfstien, 2019; Schuster et al., 2005) to define when the aridification began.

(4) $$\eta =\frac{H}{T}$$

## 4 Results

### 4.1 Fluvial Ridges on the Tushka Plain

#### 4.1.1 Morphology

**Figure 5:**
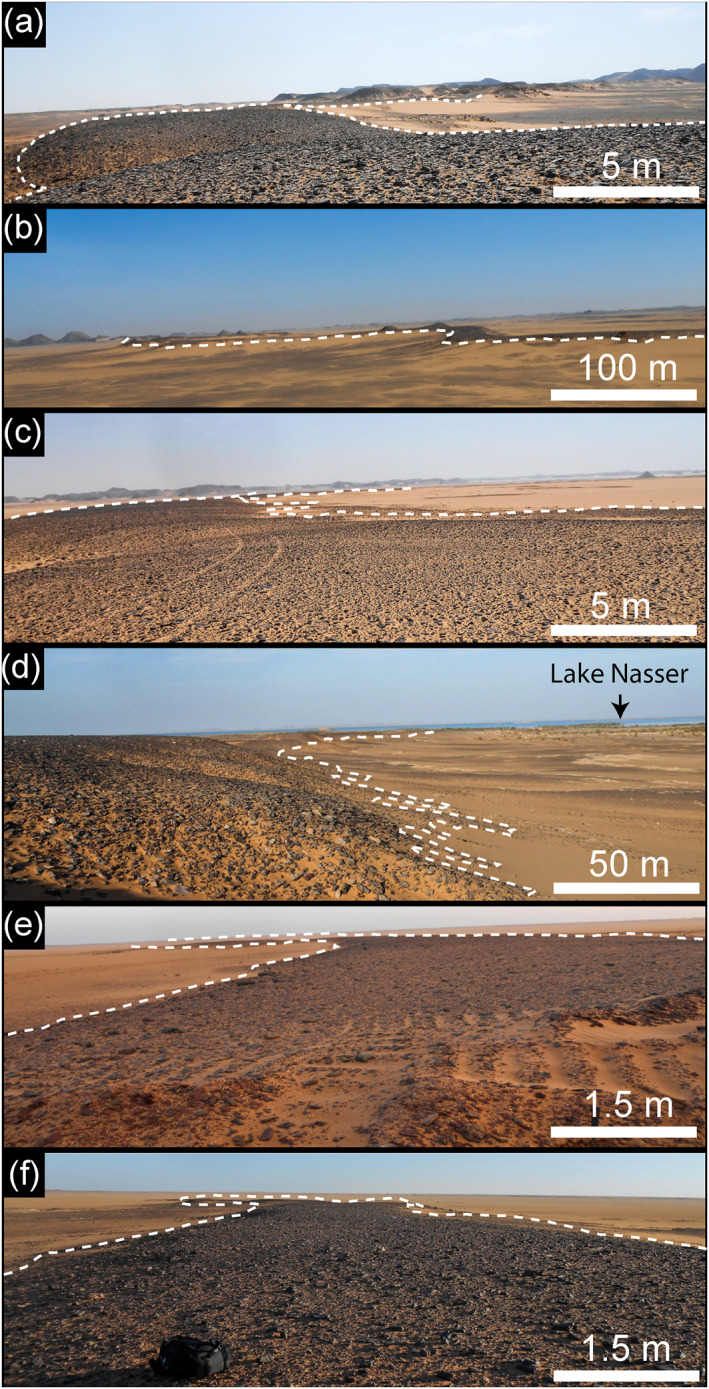
Field photographs acquired in August 2018 for the six paleorivers: (a) Gabal Hamam I (22°56'15.76"N; 32° 7'20.04"E), (b) Gabal Hamam II (22°55'17.51"N; 32° 4'25.82"E), (c) Gabal Masmas (22°51'15.28"N; 31°55'29.56"E), (d) Gabal El Sadd (22°37'30.86"N; 31°47'7.19"E), (e) Arqin I (22° 2'26.24"N; 31°12'29.75"E), and (f) Gabal Arqin II (22° 2'32.89"N; 31°16'41.35"E).

**Figure 6:**
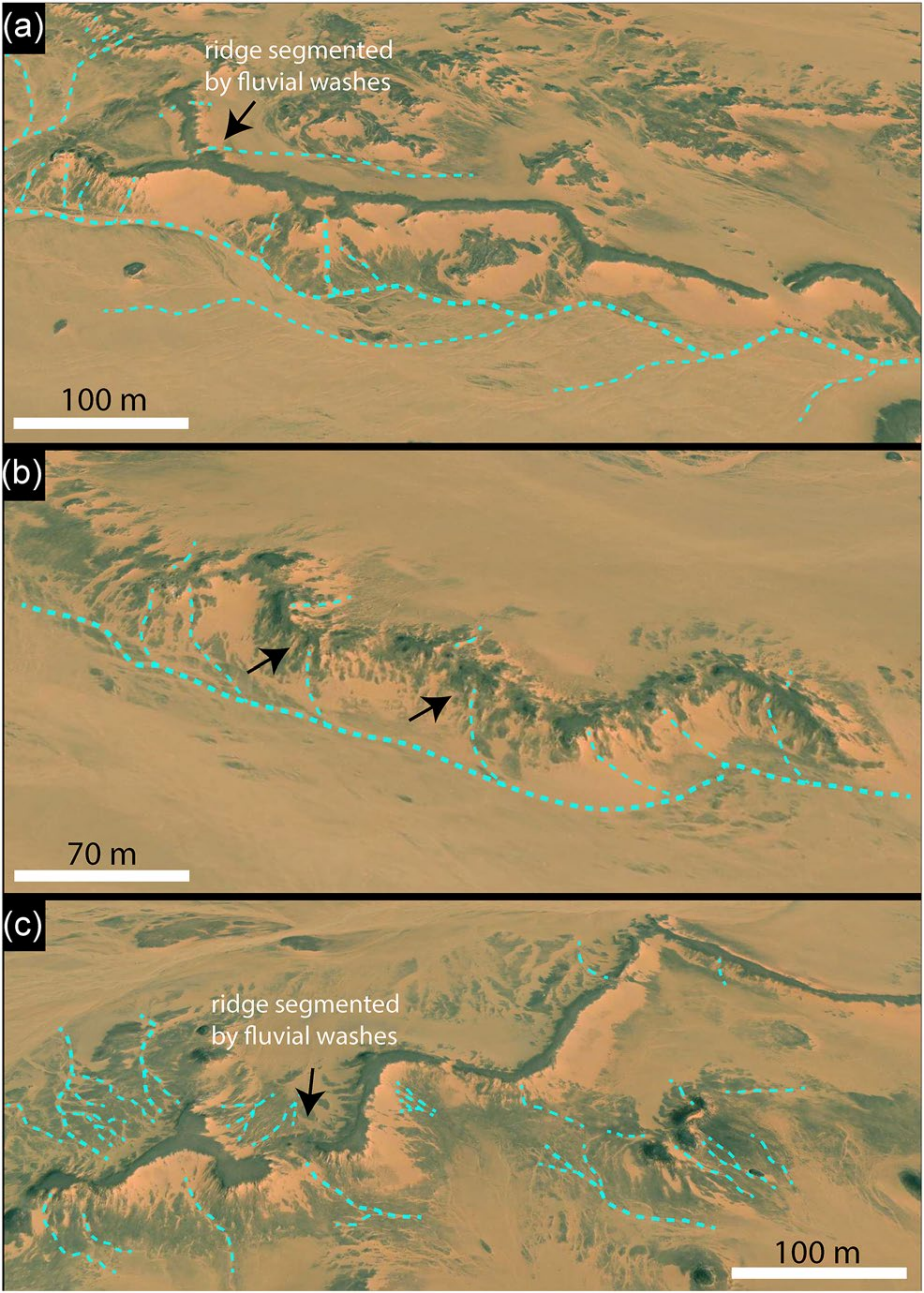
Oblique views show how the ridges were segmented by recent drainage lines responding to erosion by rainfall and surface runoff; (a) and (b) Gabal Hamam I paleoriver. (c) Gabal Hamam II. The images were derived from Esri World Imagery.

**Figure 7:**
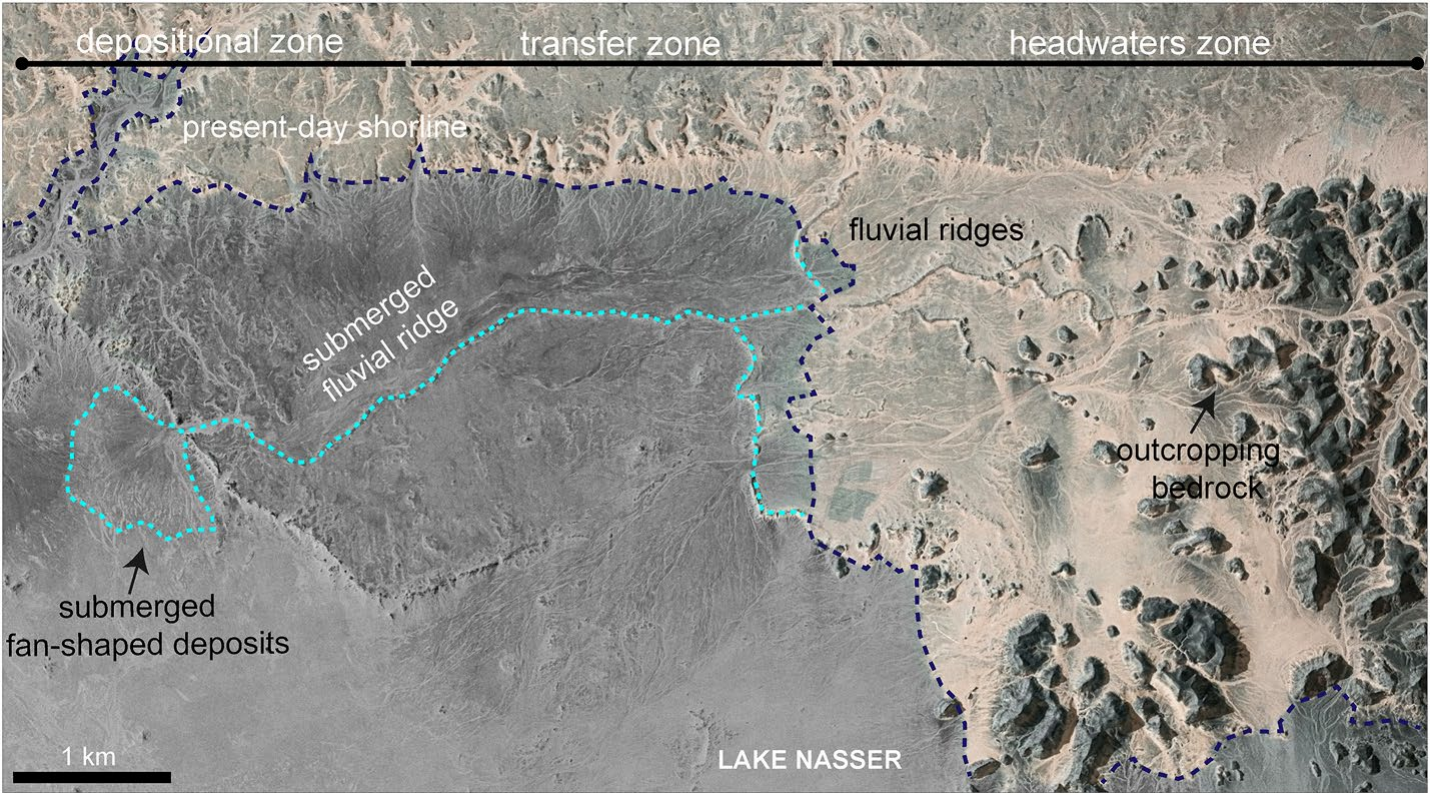
A trace of fluvial ridges in Gabal El‐Sadd paleoriver shows that these ridges flowed a short distance from source to sink. Because the sink is now submerged beneath the water of Lake Nasser, we coupled CORONA image (ID: ds1107‐1074da120) with Esri world imagery.

**Figure 8:**
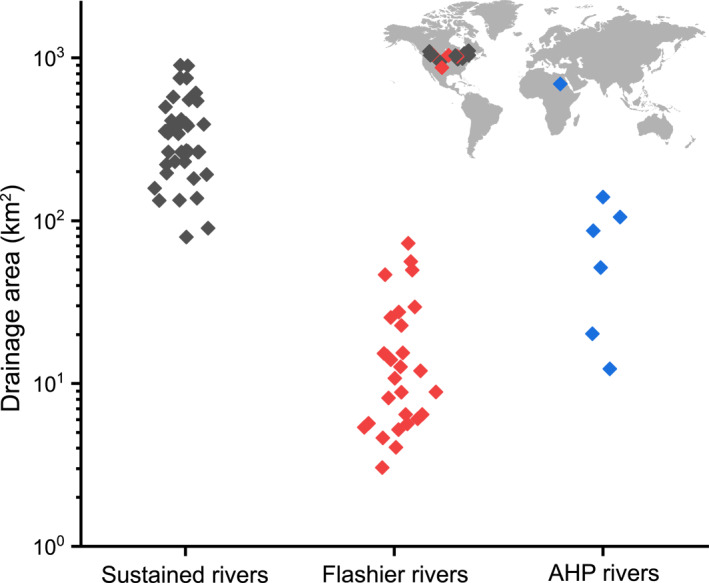
Values for drainage areas for both modern rivers survive under sustained and flashier fluvial environments and the Saharan paleorivers formed during the AHP. The figure shows that smaller drainage areas with relatively similar discharge tend to be formed under intense flash floods and the larger ones require sustained fluvial flows. The sustained rivers were compiled from (Church & Rood, 1983; Trampush et al., 2014) and flashier rivers (Patton & Baker, 1976).

The six ridge systems investigated here (Figures 4 and 5) exhibit dendritic drainage patterns in plan view. They are discontinuous, flat or convex‐topped ridges from ∼1.5 to ∼13 m high, and their widths do not exceed 65 m (Figure 5; Table S1 in Supporting Information S1). The ridges were sculpted by either water or wind, but the fluvial washes and the modern drainage lines suggest that they were segmented due to rainfall and surface runoff erosion (Figure 6). The sinuosity of the ridges ranges from 1.1 to 1.4. Most of the ridges record single‐thread channels without any evidence of existing point bars or braided stretches. The geometry of the surviving remnants of these stream systems suggests they record local drainage networks that collected water and sediment from small watersheds drained into the Nile, except for the Gabal El‐Sadd paleoriver, which debouched into a low‐relief plain on which is preserved a small fan‐shaped deposit (Figure 7 and Figure S2b in Supporting Information S1). The small‐scale geometry of this fan suggests a lack of significant sediment transport and the intermittency of the fluvial events (Figure 7). The distribution of the fluvial ridges is restricted to the areas of existing outcrops of sandstone of the Nubia Formation, suggesting that that unit is the only surficial rock unit that enables the required sequence of geologic processes to occur. The distance from the source to the sink is relatively short, ranging from ∼6 to ∼27 km in the six examples studied. Widely separated, poorly developed, and small drainage areas characterize the fluvial ridges, estimated empirically to range from 12.3 to 139 km^2^. Based on ridge widths and using (Equation 2), we estimate the discharge at 16 locations along with the six ridge sites at 33.4 ± 9.7 m^3^/s and does not exceed 245 ± 71 m^3^/s (Table S1 in Supporting Information S1). When we compared our paleorivers discharge to similar values from modern rivers surviving under different environments, we found that such discharge values require similar drainage areas under intense flash floods and larger drainage areas formed by sustained rainfall (Figure 8).

#### 4.1.2 Internal Architecture

**Figure 9:**
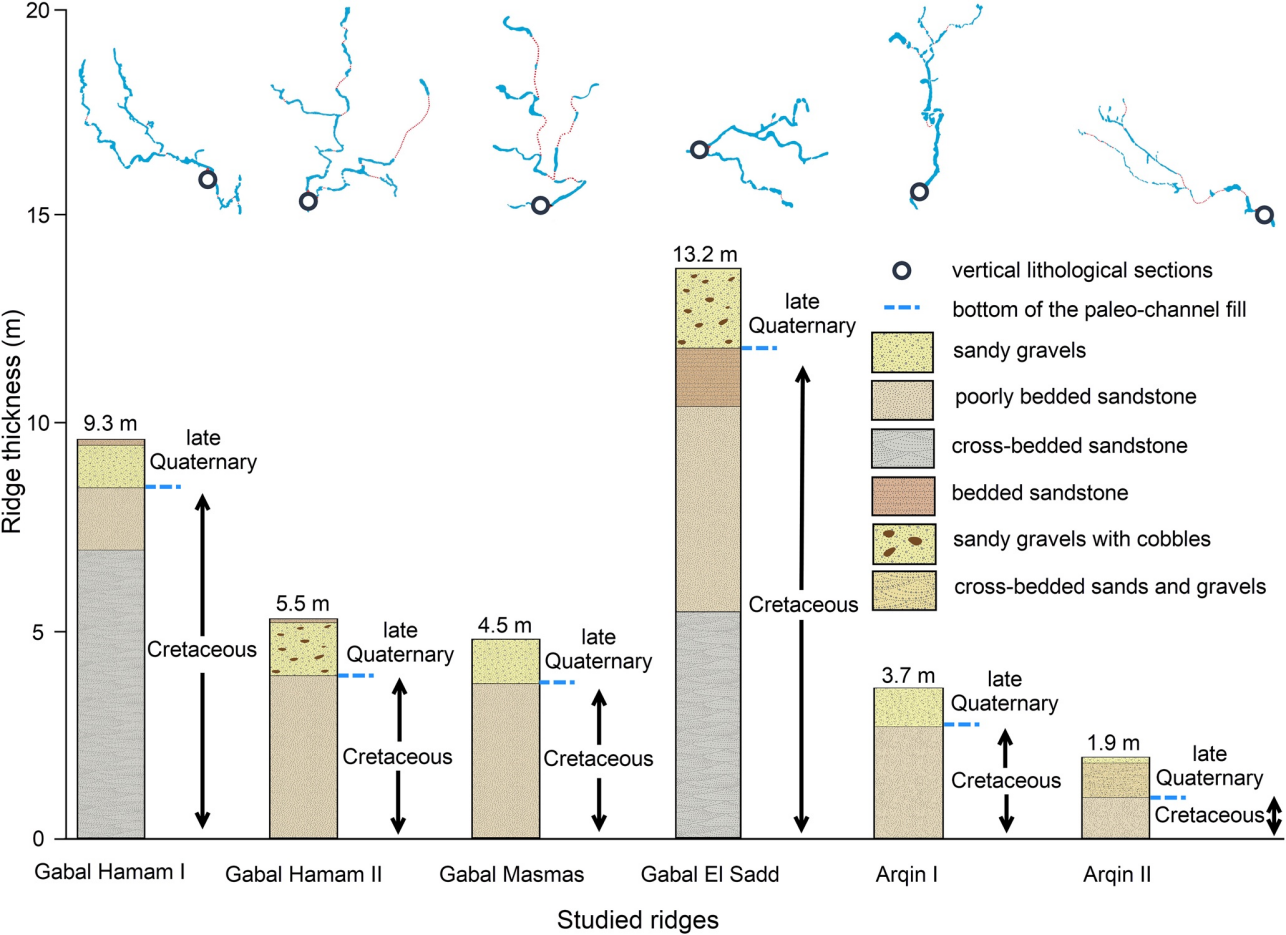
Vertical lithological sections of six of the fluvial ridges. Gabal Hamam I (22° 56′ 00″N; 32° 08′ 24″E), Gabal Hamam II (22° 54′ 39″N; 32° 04′ 13″E), Gabal Masmas (22 50 56.99N; 31 54 58.01E), Gabal El Sadd (22° 37′ 30″N; 31° 47′ 08″E), Arqin I (22° 01′ 52″N; 31° 12′ 19″E), and Arqin II (22° 02′ 33″N; 31° 16′ 39″E). The red dotted lines of the river sketches represent the missing sections.

**Figure 10:**
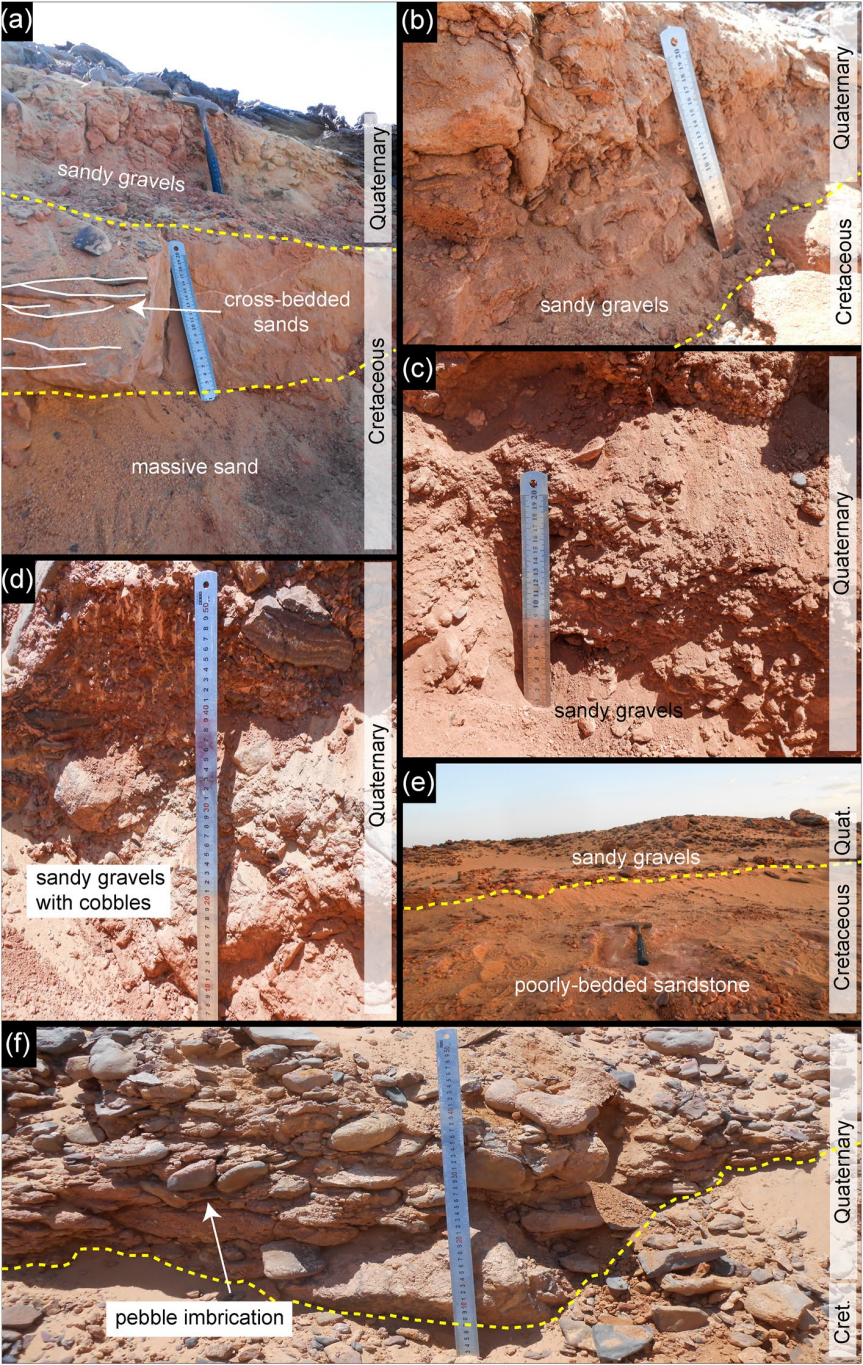
Field photographs showing examples of sedimentary structures of the fluvial ridges: (a) and (c) A side view of the Gabal Hamam I site (22° 56′ 00″N; 32° 08′ 24″E). (b) Arqin I (22° 01′ 52″N; 31° 12′ 19″E). (d) Gabal Hamam II (22° 54′ 39″N; 32° 04′ 13″E). (e) Arqin II (22° 02′ 33″N; 31° 16′ 39″E). (f) A front view of the Arqin II; ruler of 50 cm length for scale (22° 02′ 33″N; 31° 16′ 39″E). The photographs show that most of the ridges consist of two stratigraphic units; the Nubian Formation (Cretaceous) overlaid by coarse clasts (Quaternary). The yellow dashed lines in the photos are the contact between the Nubian Formation and the coarse clasts materials that were deposited during the late Quaternary. The boundary was set based on the information reported about the Nubia Formation composition.

Through investigation at 16 locations along the six fluvial ridges (Table S1 in Supporting Information S1; Figure 4), we find that the ridges are composed of two stratigraphic units. The basal unit is the Cretaceous Nubia Formation; which is overlain by Pleistocene and Holocene sandy gravels (Figures 9 and 10).

The Nubia Formation underlying the ridges consists of 0.75–12 m vertical thickness of simple cross‐bedded and planar‐bedded sedimentary quartzite derived from igneous and metamorphic rocks of the African Shield. The clasts range in size from very fine gravel (∼3 mm) to silt. The roundness of the particles varies from well‐rounded to sub‐rounded. Most of the unit is cemented by iron oxide, with a marginal contribution of the calcite, responsible for variable resistance to erosion. The clear sedimentary structures displayed in many outcrops, coupled with particle roundness and their petrography, support the inference that the Nubia Formation in this area is well‐developed alluvial sediment consistent with the interpretation of McKee (1963), who pointed out that the Nubia Formation sandstone accumulated in Upper Cretaceous time as a floodplain and lacustrine deposit. Petrographic observations of this unit (Figure 10) do not show evidence of significant compaction, including fractures and breakages, supporting the inference that the rock unit was not deeply buried, but it might have been affected by shallow burial.

**Figure 11:**
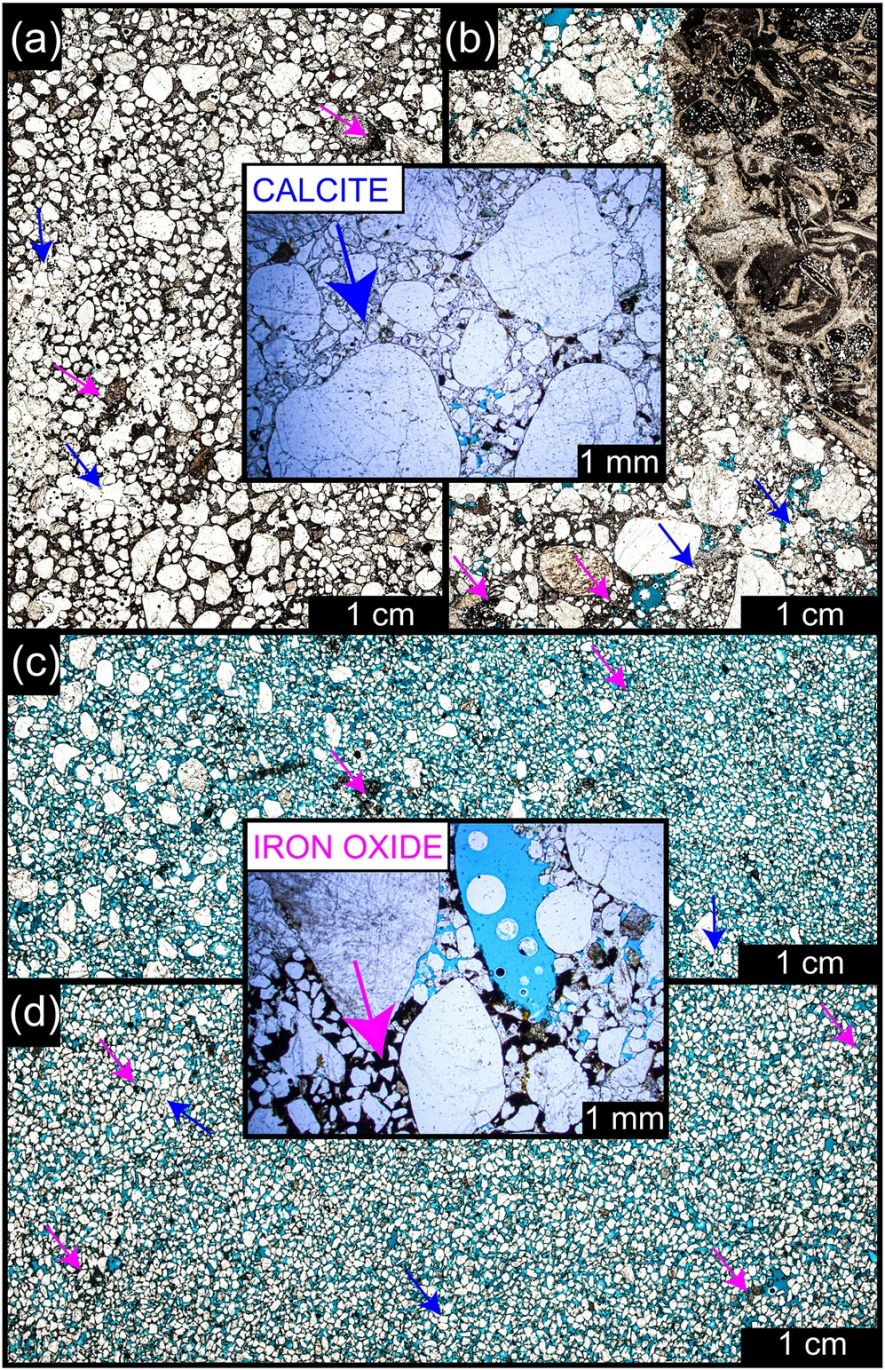
Photo micrographs showing variability in the microfacies (purple arrows indicate iron oxide and blue arrows indicate calcite; blue epoxy in pore spaces). (a) and (b) Moderately and poorly sorted sandstone with low porosity and coarse sands and gravels. (c) Moderately sorted, medium sand‐sized sandstone, with sub‐angular shape. (d) Well sorted sandstone, with fine‐grain size, ranging from angular to sub‐angular in the shape. The concretion of iron‐bearing cement likely indicates a fluvial environment. (a) and (b) collected from the late Quaternary facies from Gabal Hamam I and Gababl El‐Sadd sites, respectively. (c) and (d) represents different facies of the Nubia Formation from the Gabal Hamam I.

**Figure 12:**
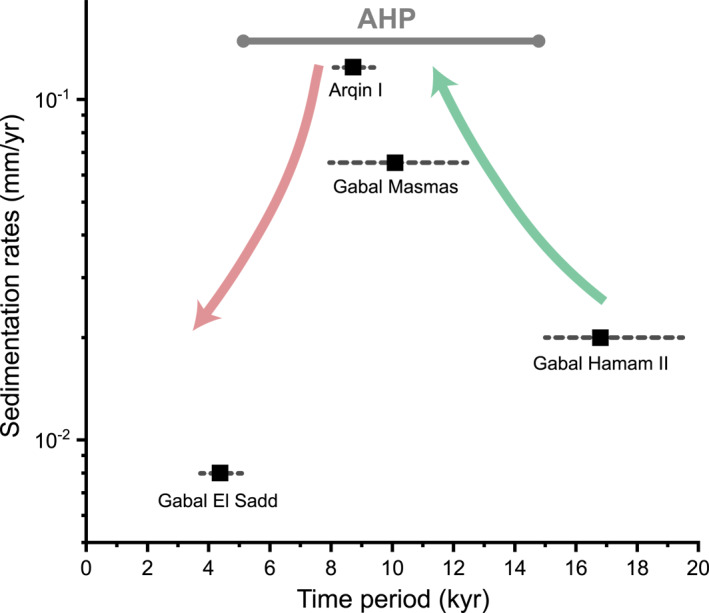
Sediment accumulation rates within four paleorivers throughout the past 20 ka. The figure shows how sedimentation rates responded to the significant increase of rainfall in Fig (3).

The sedimentary structure of the sandy gravels and the substrate suggests that these ridges represent stream channels incised into Nubia Formation sandstone. The gravel bodies are poorly sorted, ranging from 0.65 ± 0.2 to 1.2 ± 0.4 m in height. The gravel unit in each of the six fluvial ridges is not bedded except at Arqin II (Figure 9). The structureless attribute (lack of bedding) in most of the ridges might be relevant to the ephemeral streams, coupled with the rapid nature of the transport rates that occurred during the late Quaternary climate oscillation where there were rapid alternating episodes of the wet and dry conditions. The median grain size (*D*_*50*_) ranges from 21 ± 2 mm to 64 ± 2 mm (coarse to very coarse gravel). The roundness of the gravel greatly varies from well‐rounded to very angular, reflecting a wide range of different environments of transport, ranging from slower transportation rates such as in Arqin II to more extreme rates as in the rest of the ridges. The gravels in the fluvial ridges were accumulated vertically with no evidence of lateral migration, reflecting channel fill as a stacking pattern. The sandy gravels are strongly cemented by iron oxide and calcite (Figure 11). The ages obtained by Zaki, King, et al. (2021) enabled us to calculate the annual accumulation of sediments and their response to the increased precipitation rates in Figure 3 at four rivers, including Gabal Hamam II, Gabal Masmas, Gabal El Sadd, and Arqin I. We find that they range from 8 × 10^−3^ to 1.25 × 10^−1^ mm/yr. These rates peaked during the AHP due to the increased precipitation (Figure 12).

#### 4.1.3 The Preservation of Fluvial Ridges

After the cementation of the fluvial sediments either by iron oxide or calcite, differential erosion eroded the adjacent slopes, leaving the channel floors standing as ridges. Here we estimate the erosion rates and denudation volumes that eroded the substrate materials. These estimates are minimum rates and volumes, as the reconstruction of the precise elevation of the former eroded surface is not possible with the fluvial ridges. In order to constrain a reasonable age for the regional aridification that represents the onset of processes that lowered the surface, we evaluated ages determined from different proxies, including fluvial ridges, pollen records, dust fluxes, lake levels, lake sediments, and human occupation (e.g., Kuper & Kropelin, 2006; Nicoll, 2004; Zaki, King, et al., 2021). We find that the recent aridification started at ∼5 ka BP. This age is consistent with the termination of the AHP at 5.2 ka BP (e.g., deMenocal et al., 2000).

**Figure 13:**
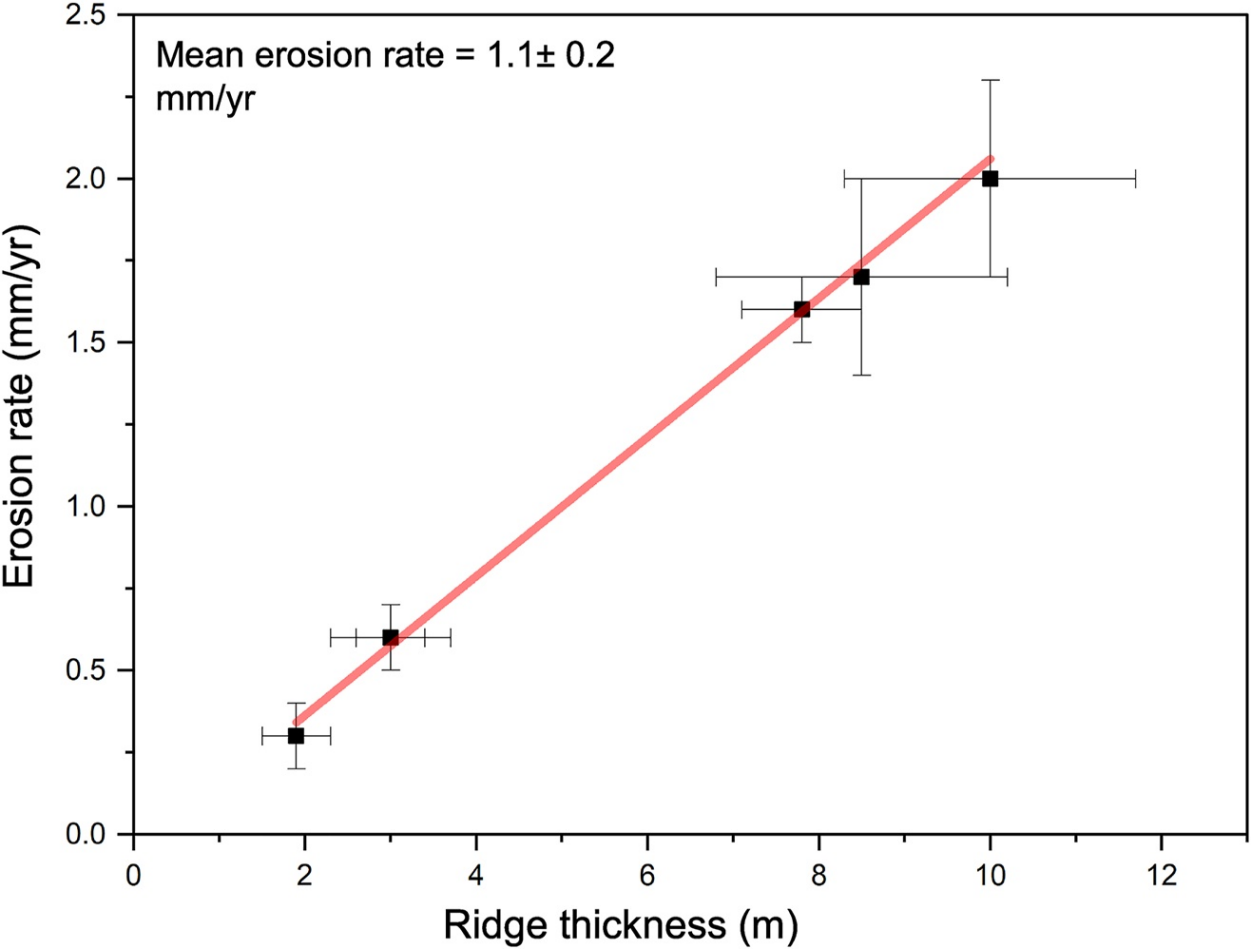
Ridge thickness and calculated erosion rates for the six paleoriver sites; Gabal Hamam I and II, Gabal Masmas, Gabal El Sadd, Arqin I and II. Each point represents the mean ridge thickness and the mean erosion rate in each site. The mean erosion rate in the whole region is ∼1.1 ± 0.2 mm/yr.

Dividing the fluvial ridge thickness at each of the six sites by the duration of aridification (∼5.2 ka) yields minimum erosion rates that range from 2 ± 0.3 mm/yr at the Gabal El‐Sadd site to 0.3 ± 0.1 mm/y at the Arqin site, with an average of 1.1 ± 0.2 mm/yr (Figure 13). The calculated volumes of eroded sediment are in the range of ∼96 ± 24 × 10^10^ m^3^, based on multiplying the average height of the fluvial ridges (5.6 ± 1.4 m) by the area in which the ridges are distributed in the west bank of the lake (17,157 km^2^). Deflation by wind and the marginal role of recent rainfall have reworked these volumes of sediment to construct various types of dunes.

### 4.2 Fan Systems in the Kiseiba‐Tushka Paleolake Basin

**Figure 14:**
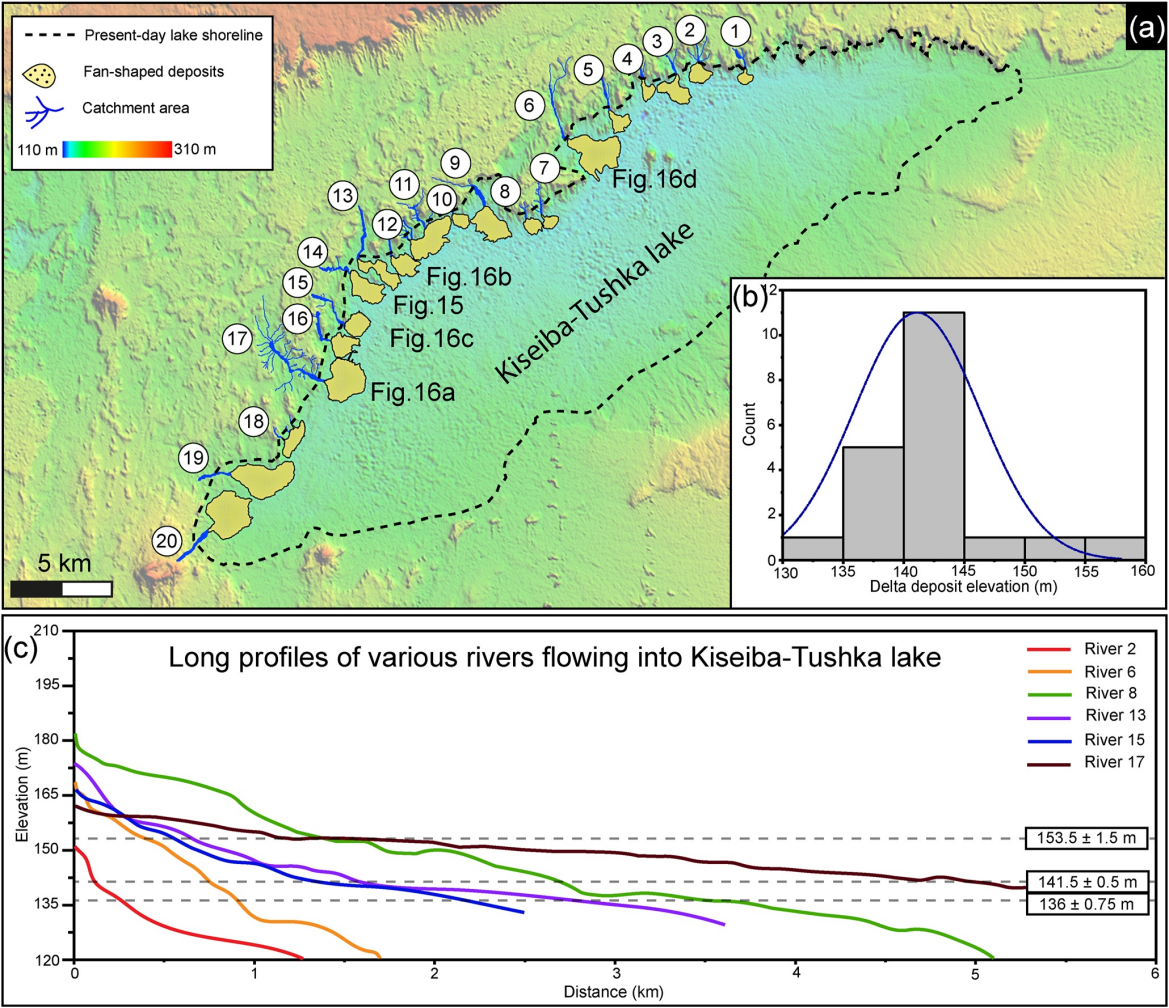
(a) Digital elevation model derived from ALOS PALSAR data shows the distribution of the deltaic features and their catchment areas. (b) Deltaic deposit elevations suggest that the progradation of most deltas took place between 140 and 145 m. (c) Long profiles of the main trunk of the rivers that drained into Kiseiba‐Tushka lake and built the fans, indicating that the accumulation of sediments occurred at multiple lake levels. The twenty rivers have been plotted in Figure S2 in Supporting Information S1.

**Figure 15:**
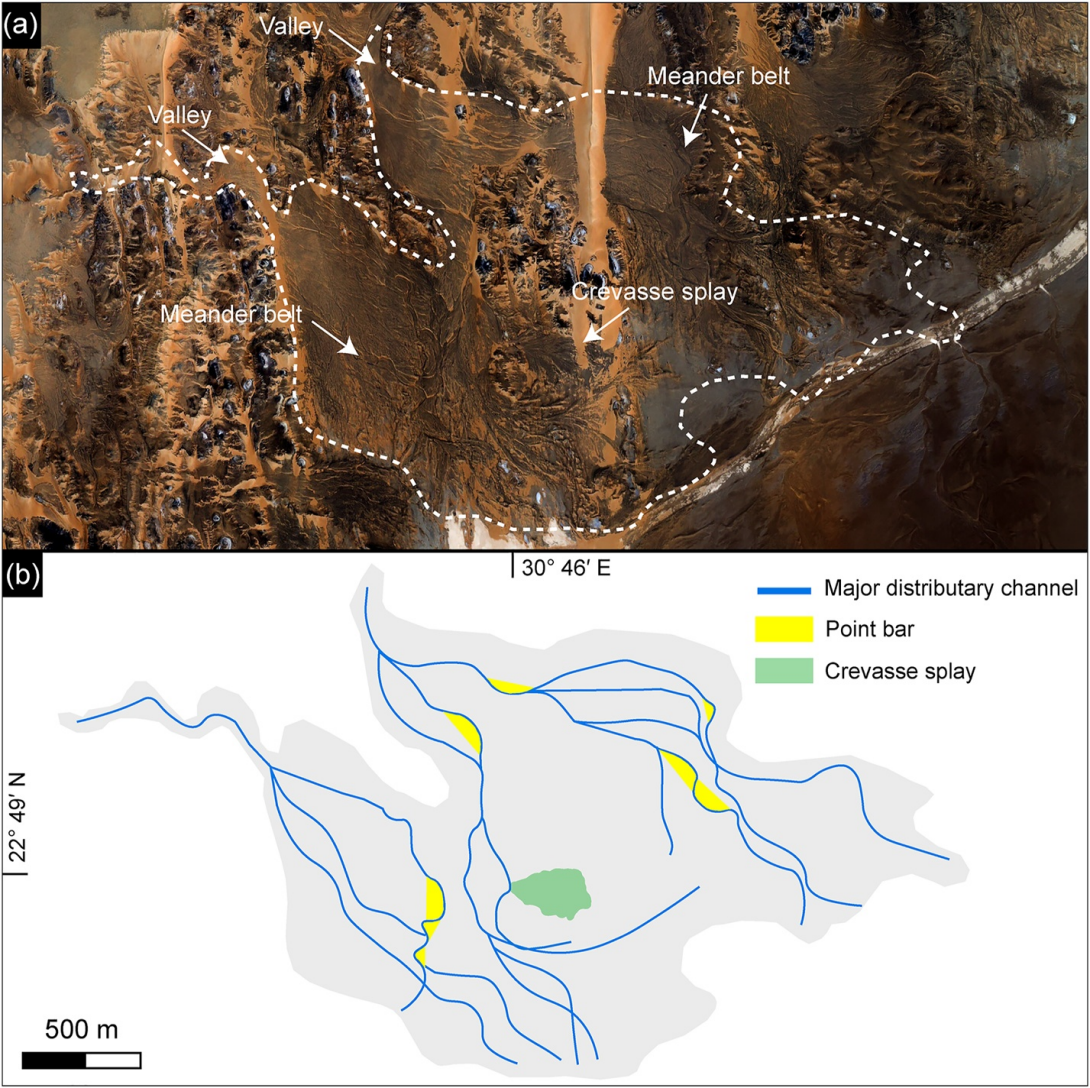
(a) and (b) Satellite image, coupled with sketch maps of main deltaic features showing the distribution of the major distributary channels, the point bars, and a crevasse splay. The satellite images were derived from Pleiades Satellite Imagery (© CNES, 2018; Distribution Airbus DS; ID: DS_PHR1A_201806180841178_FR1_PX_E030N23_1001_02095; acquisition date: 18/06/2018); 22°49'13.24"N; 30°47'11.82"E.

**Figure 16:**
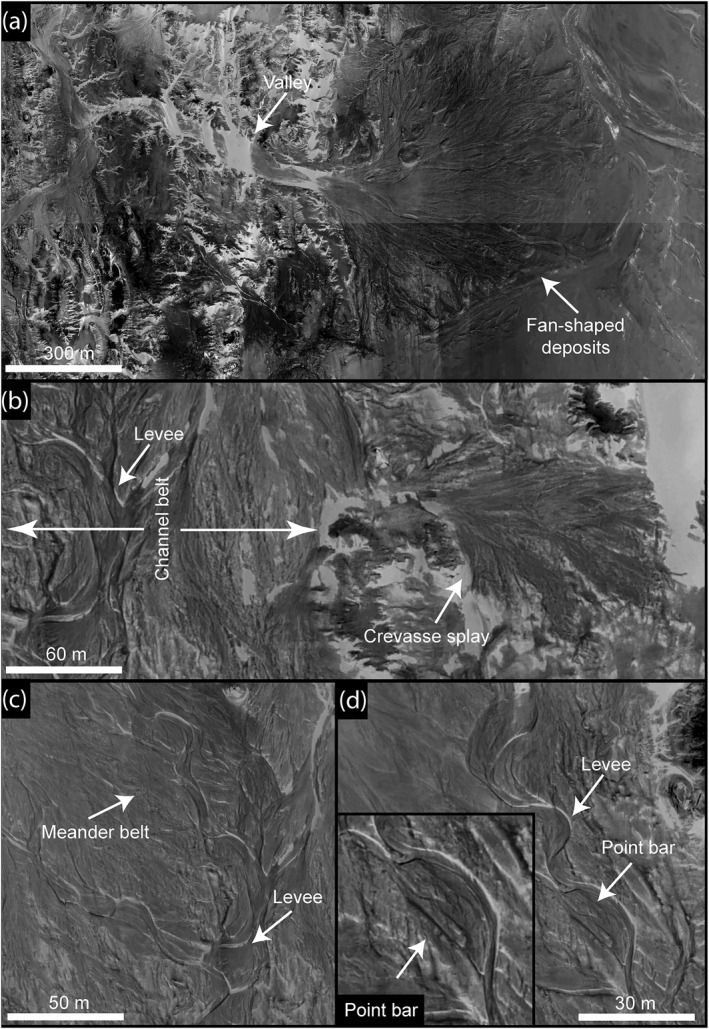
(a) Well‐preserved fan‐shaped deposits in relief inversion setting within Kiseiba‐Tushka paleolake. (b), (c), and (d) Close‐up images show the main geomorphic structure within the deltaic features, including meanders, point bar, levees, and crevasse splay. North is up in all the images. The images were obtained from Pleiades Satellite Imagery (© CNES, 2018, Distribution Airbus DS; ID: DS_PHR1A_201806180841178_FR1_PX_E030N23_1001_02095).

Our preliminary investigation from the remotely sensed data shows that twenty fan‐shaped forms that terminate in the ∼35 m–deep Kiseiba‐Tushka depression, which we have been mapped (Figure 14). They are concentrated on the north and west sides of the depression, where the catchment areas and the accommodation space were sufficient to form such fans. Some parts of these fans are relatively eroded, standing in relief in the modern landscape. Planform views show structures indicative of meanders, point bars, and splay deposits (Figures 15 and 16). Such features commonly form within floodplains. Furthermore, we identified channels bordered by levees, which could reflect development within a waterbody during base‐level changes (Figures 14b–14d). The low‐relief depressions in the region, which encapsulates all of the features, was also identified by Nicoll (1998) as a playa lake that was likely active during the early to mid‐Holocene time. Thus, we consider these fans‐shaped deposits to be deltas rather than alluvial fans.

These deltas have small surface areas ranging from 0.16 to 1.71 km^2^ (Table S2 in Supporting Information S1). Using ALOS PALSAR DEM, we obtained their present‐day slopes that do not exceed 0.014, and each presents a radial fluvial distributary pattern. When considering the varying elevations of the fan deposits, we see three distinct elevations at which the slope changes in the measured long profiles of the deposits. This indicates that these deltas fanned out into the paleolake at three different lake levels which are today at 136 ± 0.75 m asl, 141.5 ± 0.5 m asl, and 153.5 ± 1.5 m asl (Figures 14b and 14c). Based on measurements of the deltas' thicknesses from DEMs, we find that the best estimate ranges 10–17 m. These deltas accumulated at the base level of the lakes from drainage areas ranging from 0.18 to 4.78 km^2^, consistent with Hack's law calculation. The trunk valleys of each catchment zone have sharp walls with theater‐head valleys in most examples, suggesting post‐incision modification by groundwater seepage, similar to those are concentrated along the Libyan escarpments (Abotalib et al., 2016). The valley trunks are tens of meters wide to a maximum of ∼200 m.

The sedimentary bodies of each delta preserve incised small streams, fluvial ridges (topographically inverted stream channel forms), and point bars (Figures 15 and 16). In 14 of the 20 deltas, point bars are dominant. They appear in planform as sets of meander scroll ridges with individual heights ranging from >1 m to <4 m. The lateral migration distances have been measured on 48 point bars and indicate 4.6–56.3 m based on the extensive downstream stacking of adjacent meander scrolls (Table S3 in Supporting Information S1). Using an empirical relationship developed by (Lapôtre & Ielpi, 2020), the migration rate ranges from 0.06 ± 0.002 m/yr to 0.63 ± 0.03 m/yr under vegetated environments, increasing with the channel width. Dividing the migration distance by the migration rate suggests a migration time span of ∼25.2 ± 0.9 to ∼187 ± 6.2 years. Crevasse splay features are observed near a meandering system in one delta (Figures 15 and 16), suggesting that the water and sediments broke the outer banks during high discharge events.

**Figure 17:**
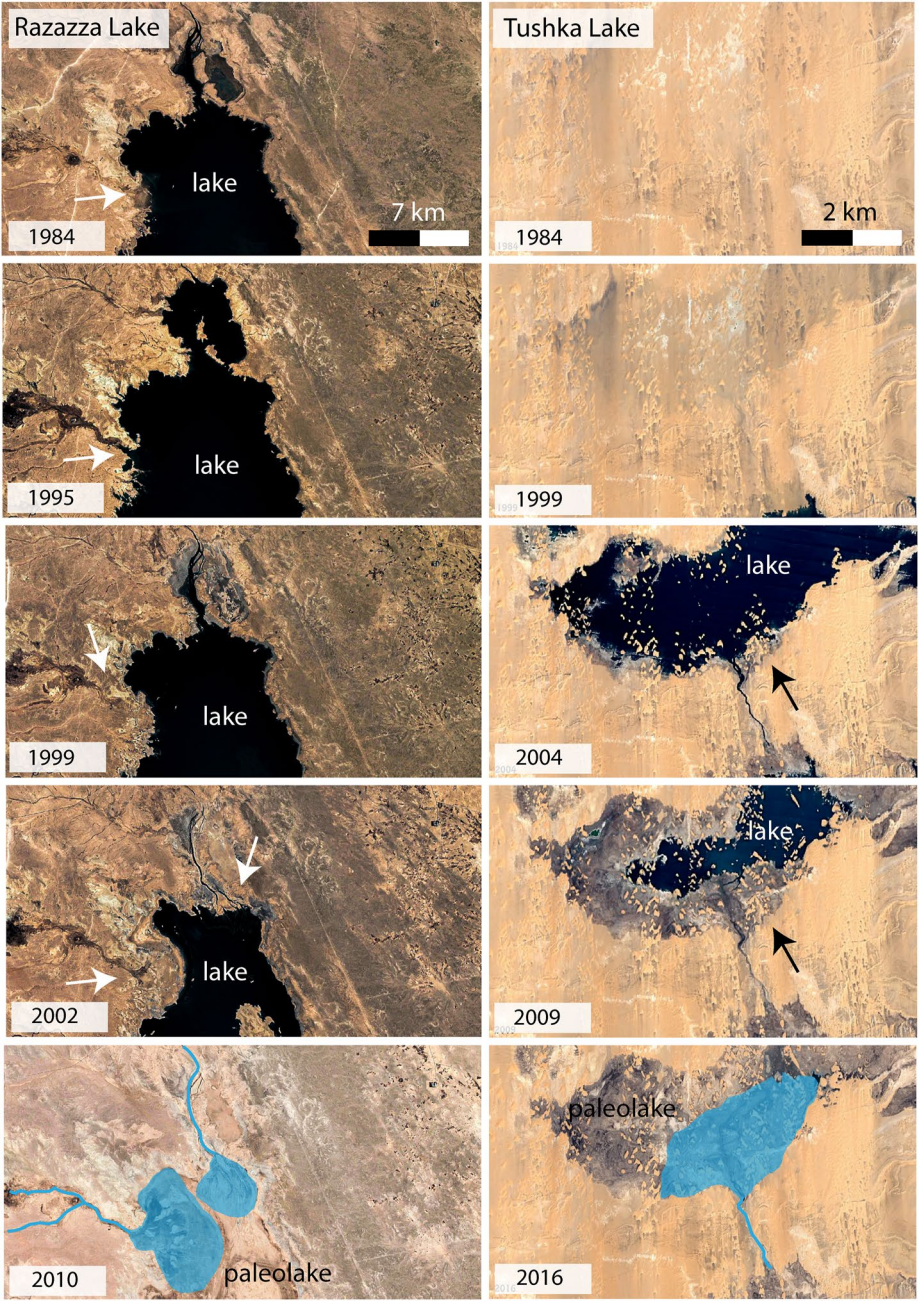
Time series of two highly fluctuating modern lakes from the Egyptian Sahara showing deltas' formation over a few decades and confirming that tens of years are sufficient to form similar deltas to those investigated here (images source: Google Earth).

Observations from satellite images for the modern analogs from Razazza Lake in Iraq and modern Tushka lake are consistent with the estimated durations, confirming that similar deltaic features require a few tens of years of continuous alluvial activity in shallow depositional environments (Figure 17). Movies S1 and S2 in Supporting Information S1, available as supporting information, also show the formation of similar deltaic features within modern lakes.

We observed many ridges that we interpret as fluvial deposits within 15 of 20 deltas (Figure 16). These ridges are well‐preserved at the distal end of the fans with a lateral extent of tens of meters to a few hundred of meters. They stand up to ∼5 m high in the modern landscape, with a maximum width of 150 m and a surface slope toward the lake. These ridges record channel belts based on their planform shape, reflecting lateral migration in only three deltas, with evidence of ridge superposition that might express vertical aggradation.

## 5 Discussion

The eastern Sahara’ fluvial systems described here provide a unique window to understand the fluvial history that controlled the eastern Sahara during more humid conditions. They preserve short‐distance locally sourced systems and consist of fluvial ridges and the deltaic features, recording a series of climatic‐driven landscape changes before, during, and after the AHP. These systems were formed by channel‐belt and channel‐fill construction by river‐channel migration and aggradation. Channel incision and sediment deposition were followed, during more arid times, by topographic inversion via differential erosion by wind and erosion by rainfall‐generated runoff. Such landforms in arid regions are instructive for our understanding of the early martian climate, as they are a snapshot of fluvial history followed by aeolian environments. Thus, the fluvial landscape in southern Egypt provides a natural laboratory to consider the paleoclimate and paleoenvironment context for the evolution of similar martian landforms, approximating multi‐phase genesis, formation, preservation, and timescale.

Our observations of the form and content of the fluvial ridges suggest that the sandy gravel deposits stacked in the fluvial ridges were accumulated in paleochannels as ephemeral streams during high energy events over fast‐responding landscapes. The upstream fluvial ridges suggest that intense precipitation events formed them through relatively high transportation rates from local sources, as inferred from the large median grain size (*D*_*50*_ = 21–64 mm), and the stacked gravels in the ridges lack internal stratification. Non‐layered (depositionally structureless) gravels lacking bedding are common in rivers worldwide, and they have been explained by rapid transportation rates that reduce the chance of the development of an armored layer, particularly under ephemeral conditions (Laronne et al., 1994). Our estimates of paleodischarge using width relationships falling in the range of 33.4 ± 9.7 m^3^/s to 245 ± 71 m^3^/s also support the high energetic flows, particularly those sourced from relatively small drainage areas. These observations and calculations are consistent with the rainfall intensities involved in forming these rivers that fall in the range of 55–80 mm/hr over timescales ranging from 10^3^ to 5 × 10^3^ years (Zaki, King, et al., 2021). Moreover, when comparing the rivers with nearly similar discharge from different fluvial depositions environments, our drainage areas are close to those formed under flashier climates (Figure 8). A similar paleoenvironmental interpretation has been observed from fluvial ridge systems in the Kumtagh Desert, China (Wang et al., 2015), Ad Dwasir, Saudi Arabia (Matter et al., 2016), and the Atacama Desert (e.g., Morgan et al., 2014; Williams et al., 2021), where coarse materials from local bedrock sources are prevalent and were deposited over short durations, that is, thousands to tens of thousands years. Hayden, Lamb, and McElroy (2021) calculated the median of intermittency of the gravel‐bed river at 0.092, which means that the formative fluvial events could occur over a few days to several weeks every year, which could be applied to the ancient gravel‐bed rivers investigated here.

The martian surface preserves multiple examples that record ancient rivers and streams are characterized by short‐distance (a few kilometers from the source to the sink), single‐thread channels like those in the western exposures of the Medusae Fossae Formation and on the plateau immediately west of Juventae Chasma on Mars (e.g., Di Pietro et al., 2018; Harrison et al., 2013; Weitz et al., 2008; Williams et al., 2007), reflecting river channel fill rather than channel‐belt accumulation like the Izola outcrop in the northwestern shoulder of the Hellas Basin (Salese et al., 2020). Our interpretation based on the terrestrial analogs, therefore, lends support to the conclusion that the single‐thread, short‐distance source‐to‐sink fluvial ridges on Mars might have resulted from local bedrock sources subject to intense rainfall, leading to high sediment‐transport rates.

Our preliminary investigation of the fan‐shaped deposits shows that they record delta progradation and retrogradation at multiple stages with falling and rising the lake level (Figure 12). This progradation and retrogradation also could imply the presence of an ephemeral water body with fluctuating water levels (Saez et al., 2007). However, further age constraints and a detailed investigation of the internal architecture of these fans are essential to confirm the lake level fluctuations hypothesis. Levee forms, coupled with crevasse splays, have been observed at many fans, supporting the episodes of intense floods (e.g., Lepre, 2017), consistent with the observation of large clasts within the ridges. There are no constraints on the deposition of these fans, but Nicoll (1998) proposed the closed basin that hosts the fans to be one of these playa lakes that were active during the last AHP (early to mid‐Holocene). To understand how sustained the fluvial activity lasted during the AHP, our estimates of migration rates and the time span required to form the meandering river systems within the deltas suggest at least 25.2 ± 0.9 up to 187 ± 6.2 years of river activity during the AHP. These durations indicate that these fluvial forms reached the bankfull channel for 1.2–8.8 days per year.

These ancient fans are also vital as a proxy for our understanding of early martian climate, since Mars has numerous fan‐shaped landforms deposited in low‐relief areas, bearing signatures of paleolakes based on existing outlet valleys and associated sedimentary rock (e.g., Fassett & Head, 2008; Grotzinger et al., 2015). The accumulated sediments in the paleolakes as relief‐inverted, fan‐shaped deposits have been considered to be deltas by multiple studies (e.g., Di Achille & Hynek, 2010; Fassett & Head, 2005; Goudge et al., 2018; Malin & Edgett, 2003). Many of these deltas are locally sourced, recording deposition in shallow lakes (e.g., Di Achille & Hynek, 2010; Irwin et al., 2015; Malin & Edgett, 2003). Our preliminary geomorphic observations from the Saharan deltaic features, coupled with migration rate estimates and chronological context of the Saharan pluvial periods, suggest that similar fans on Mars might have formed under relatively wet conditions that spanned tens to a few hundred years over a total duration of thousands of years, in agreement with current literature (e.g., Kleinhans, 2005; Lapôtre & Ielpi, 2020; Stucky de Quay et al., 2019). Such estimates suggest rapid sediment aggradation and burial, making the deltas an instructive site for testing if life ever existed on early Mars, as they may preserve organic matter because of rapid burial (Ehlmann et al., 2008; Goudge et al., 2018; Lapôtre & Ielpi, 2020). Furthermore, the fan deposits would have concentrated fine‐grained materials that are favorable to preserving organic materials (Mangold et al., 2020).

Because of the possibility of organics being carried by water, fluvial deposits have been considered as a possible biosignature repository on Mars (Summons et al., 2011). This information is encoded in both the modes and durations of depositional history. Hayden et al. (2019) suggested that ridges recording long‐lived fluvial activity that persisted over millions of years may favor habitability on early Mars. However, Williams et al. (2021) reported fluvial ridges from the Atacama Desert, Chile, preserving potential biosignatures, including plants and subsurface organisms over short durations, that is, thousands of years. This study also indicates that fluvial landforms deposited by short‐duration fluvial activity (10–100s years over thousands of years) also supported habitable environments for biosignature, including bacterial structures and organisms in the eastern Sahara (e.g., Cremaschi et al., 2010; Nicoll & Sallam, 2017). This suggestion makes such sediments scientifically very important for future sample return missions.

Because detailed measurements of sedimentary structures within the martian ridges are not feasible using orbital images, improving paleo‐hydraulic methods that depend on ridge widths is important to constrain the paleodischarge better. Our estimates from the width‐based discharge fall in the range of 33–245 m^3^/s. These estimates differ from discharge quantified from grain‐size distribution and paleochannel channel geometry (43–138 m^3^/s; Zaki, King, et al., 2021). When comparing the two values quantified from the two methods, the width‐discharge relationship yields a factor of ∼70% higher than the discharge estimated from the grain size and slope reconstruction. The low discharge rate quantified from grain‐size distribution and paleochannel geometry could be the minimum rate since there are uncertainties on grain‐size measurements, and the erosion has played an important role in eroding the original channel geometry. However, this value could help when estimating the discharge based on width from orbital images.

**Figure 18:**
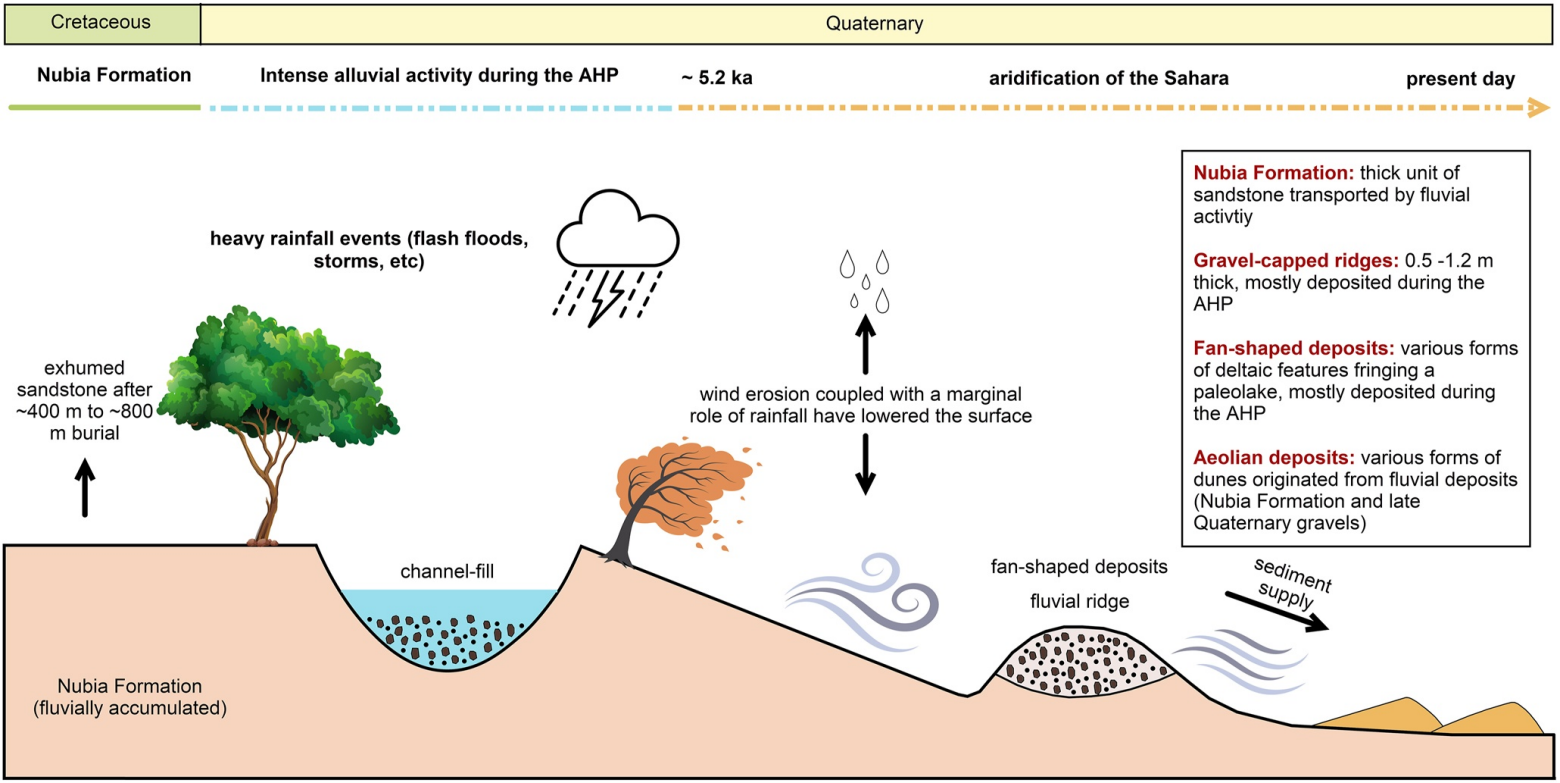
Schematic diagram showing the transition from fluvial activity to aeolian processes in southern Egypt.

After the termination of the last pluvial period in the Sahara at ∼5.2 ka, the region dried up, and the aeolian processes, including deflation and wind abrasion (including occasional flashy rainfall events; Figure 18), lowered the surface at least 1.5–13.2 m, providing sediment some of which accumulated as various forms of dunes. Ridge thickness coupled with the age aridification enabled us to quantify the minimum erosion rates and denudation volumes involved in preserving the fluvial systems in the region. The calculated erosion rates are in the range of 0.3 ± 0.1 to 2 ± 0.3 mm/yr^−1^, and the denudation volume of the eroded sediments is ∼96 ± 24 × 10^10^ m^3^. These volumes are nearly three times more than those eroded from the inverted fans in Oman (Mazeiles, 1987). The wide range of erosion rates (0.3–2 mm/yr) might be relevant to the consolidation control (Rohrmann et al., 2013). The consolidation variability of the Quaternary deposits that were never buried nor exhumed differs significantly from the Nubia Formation that buried, lithified, then exhumed. Similar ridges and modeled ages from crater counting can be used on the martian surface as a proxy to estimate the minimum erosion/exhumation rates, providing a better understanding of landscape evolution since the arid conditions of modern Mars replaced wet conditions that prevailed on early Mars.

Overall, the AHP event offers a time‐bounded, well‐constrained narrative of environmental and climatic changes from wet to arid conditions that may exemplify the dramatic climate changes that Mars experienced in its early history. Our investigation of locally sourced fluvial depositional systems developed during the AHP provides glimpses into the formation and preservation of similar forms on Mars, suggesting that local and transient fluvial activity might have punctuated early martian climate over short‐duration events. Alongside the martian ridges that might record fluvial activity over prolonged periods of geologic time (∼10^5^–10^6^ years; e.g., Davis et al., 2019; Hayden et al., 2019; Balme et al., 2020; Cardenas et al., 2020; Hayden & Lamb, 2020; Stucky de Quay et al., 2019), our results show that other ridges on Mars may have formed in brief, ephemeral events, over periods as short as a few thousand years. We emphasize that caution should be used when interpreting fluvial landforms from orbit due to this convergence of form. Taken together, our results add to the growing recognition of the potential of fluvial depositional systems for constraining the water and wind driven activities that might have dominated on early Mars.

## 6 Conclusions

The eastern Sahara shows a series of locally sourced, fluvial depositional systems, now expressed as sinuous to branching ridges in the modern landscape. They are widely distributed across ∼38,000 km^2^, extend for tens of kilometers, are as much as 65 m wide, and as high as 13 m. The ridges are discontinuous, representing fragments of dendritic drainage patterns. The internal structure of these ridges records two main stratigraphic units: Cretaceous Nubia Formation accumulated by fluvial activity, overlain by cemented sandy gravels fluvially deposited during mostly the AHP. The AHP gravel beds do not exceed 50% of the whole ridge thickness. Internally, the structure of the gravel beds displays non‐layered gravels with angular to subrounded clasts ranging from 21 to 64 mm in grain size (*D*_*50*_). The architecture of the gravel beds suggests that they were deposited by ephemeral rivers formed in response to intense precipitation and rapid sedimentation rates over rapidly responding landscapes throughout short durations (10–100s years over ∼10,000 years).

Some of the fluvial ridges are well preserved within fan‐shaped deposits that we interpret as deltaic features. The minimum duration required to form the meandering system within the deltas spanned ∼25–187 years, most likely were active throughout the AHP for ∼10,000 years. The deltas were sourced from local small drainage areas, and their formation took place during at least three stages of lake‐level fall and rise. Further investigation of the distribution, sedimentary architecture, age constraints of these fans would offer the possibility of understanding both the past climatic conditions that persisted during the Saharan pluvial periods.

The fluvial ridges have been eroded by both wind and water, forming ridges; we estimate the minimum erosion rates to be in the range of 2 ± 0.3 mm/y to 0.3 ± 0.1 mm/y. Our best estimate for the volume of eroded sediments is 95,000 ± 25,000 km^3^; some of that volume has been reworked by the wind to form various forms of dunes, including linear, lee, and barchan dunes.

Locally sourced martian fluvial ridges that record short distance source‐to‐sink channels might have been formed from ephemeral streams fed by intense fluvial activity that took place over short durations, that is, thousands to a few tens of thousands of years. Our observations may imply that the early martian climate could have been punctuated by short periods of locally high precipitation that formed short source‐to‐sink ephemeral channels and rivers. Partial erosion of such ridges during the transition from wet to dry conditions might have provided sufficient sediment to contribute to the formation of the aeolian dunes on Mars.

## Supporting information

Supporting Information S1

Figure S1

Figure S2

## Data Availability

Supporting information includes Figure S1 in Supporting Information S1 processed from Landsat 8 satellite imagery, accessible from the following website (Irons et al., 2012; https://earthexplorer.usgs.gov/). Table S1 includes raw data collected in the field of fluvial ridge locations, measurements, and discharge calculations. Tables S1, S2, and S3 in Supporting Information S1 represent measurements obtained from remotely sensed data, including Esri WorldImagery (https://www.arcgis.com/home/), and ALOS PALSAR DEM (Rosenqvist et al., 2007) archived here (Zaki, 2021; https://doi.org/10.5281/zenodo.6319858). The CTX and HiRISE images (Malin et al., 2007; McEwen et al., 2007) that have been used in preparing Figure 1 are also archived here (Zaki, 2021; https://doi.org/10.5281/zenodo.6319858).The Movies S1 and S2 in Supporting Information S1 were created via images from Google Earth historical imagery (https://earthengine.google.com/timelapse/). Pléiades' images (Lebegue et al., 2012) used in this study are not publicly available for commercial reasons but can be purchased from the following website (https://www.intelligence-airbusds.com/imagery/constellation/pleiades/).
