# Supplementary material for: Fluvial Depositional Systems of the African Humid Period: An Analog for an Early, Wet Mars in the Eastern Sahara
Source: J Geophys Res Planets. 2022 May 13;127(5):e2021JE007087. doi: 10.1029/2021JE007087 (PMC9285406; doi:10.1029/2021JE007087)
Supplement: Supplementary file 1 — Supporting Information S1 [file JGRE-127-0-s001.pdf]

AGU PUBLICATIONS

Journal of Geophysical Research Planets

Supporting Information for

Fluvial depositional systems of the African Humid Period: An Analog for an Early, Wet Mars in the Eastern Sahara

A. S. Zaki[1*], J. M. Davis[2], K.S. Edgett[3], R. Giegengack[4], M. Roige[5], S. Conway[6], M. Schuster[7], S. Gupta[8], F. Salese[9,10], K.S. Sangwan[8], A. G. Fairén[9,11], C.M. Hughes[12], C.F. Pain[13], and S. Castelltort[1]

[1]Department of Earth Sciences, University of Geneva, Rue des Maraîchers 13, 1205 Geneva, Switzerland

[2]Department of Earth Sciences, Natural History Museum, Cromwell Road, Kensington, London, SW7 5BD, UK

[3]Malin Space Science Systems, Inc., P.O. Box 910148, San Diego, CA 92191, USA

[4]Department of Earth & Environmental Science, University of Pennsylvania, Philadelphia, PA, USA
[5]Departament de Geologia, Universitat Autònoma de Barcelona, 08193 Bellaterra, Spain.
[6]CNRS UMR 6112 Laboratoire de Planétologie et Géodynamique, Université de Nantes, France
[7]Université de Strasbourg, CNRS, Institut Terre et Environnement de Strasbourg, UMR 7063, 5 rue Descartes, Strasbourg F-67084, France
[8]Department of Earth Sciences and Engineering, Imperial College London, London, SW7 2AZ, UK
[9]Centro de Astrobiología (CSIC-INTA), Torrejón de Ardoz, Madrid, Spain
[10] International Research School of Planetary Sciences (IRSPS), Università d'Annunzio, Viale Pindaro 42, 65127, Pescara, Italy
[11]Department of Astronomy, Cornell University, Ithaca, NY, USA
[12]Department of Geosciences, University of Arkansas, Gearhart Hall, 340 N Campus Dr, Fayetteville, AR 72701, USA
[13]MED_Soil, Departamento de Cristlografía, Mineralogía y Quimica Agrícola, Universidad de Sevilla, 41012 Sevilla, Spain.

Contents of this file

Figures S1 to S2
Movies S1 to S2

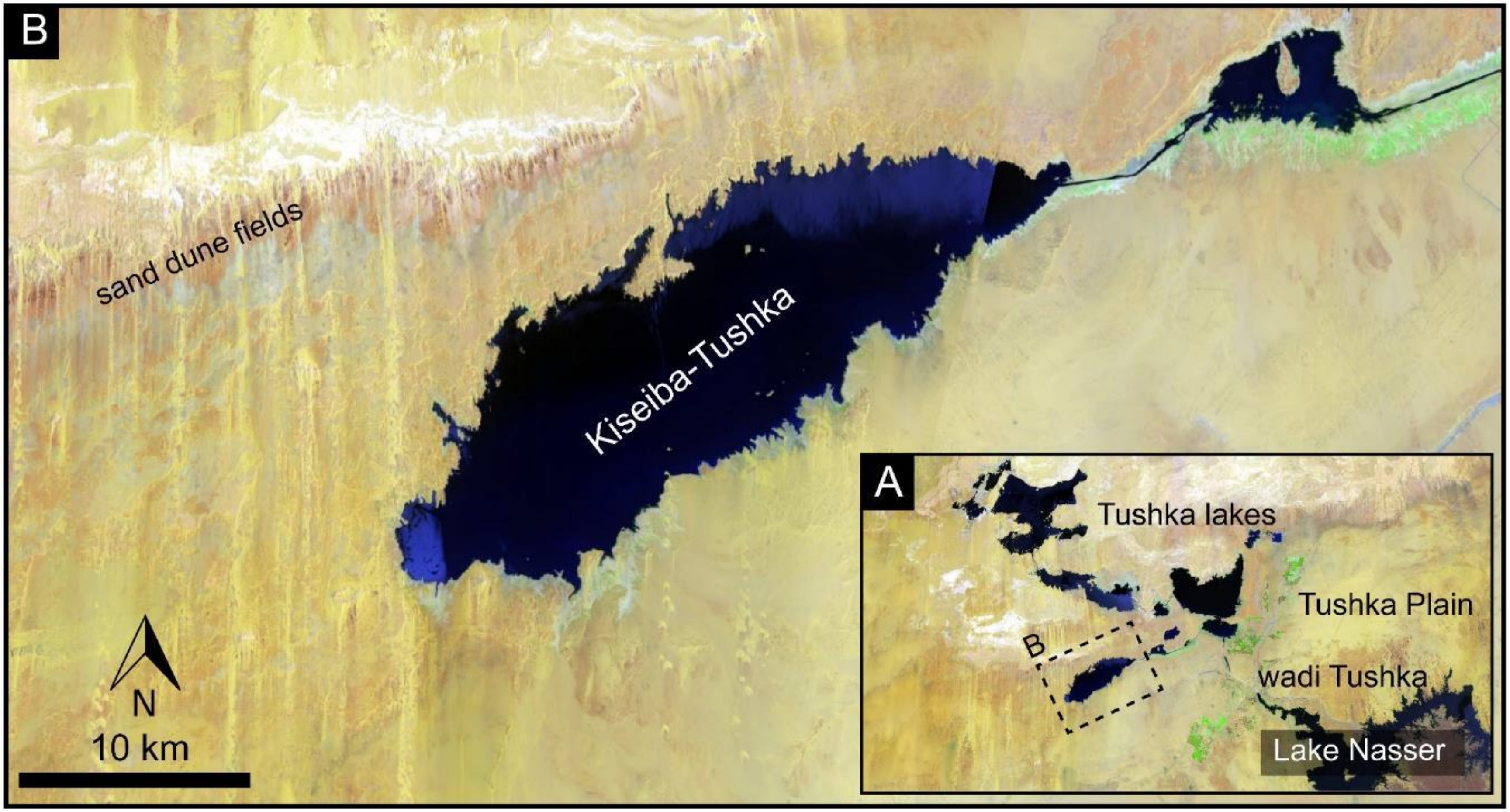

Figure S1. (A) Landsat mosaic showing the location of the Tushka paleolakes. **(B)** Present-day Kiseiba-Tushka lake. The fan-shaped deposits are now submerged beneath the water drained from Lake Nasser. (Landsate imagery – 28/06/2021).

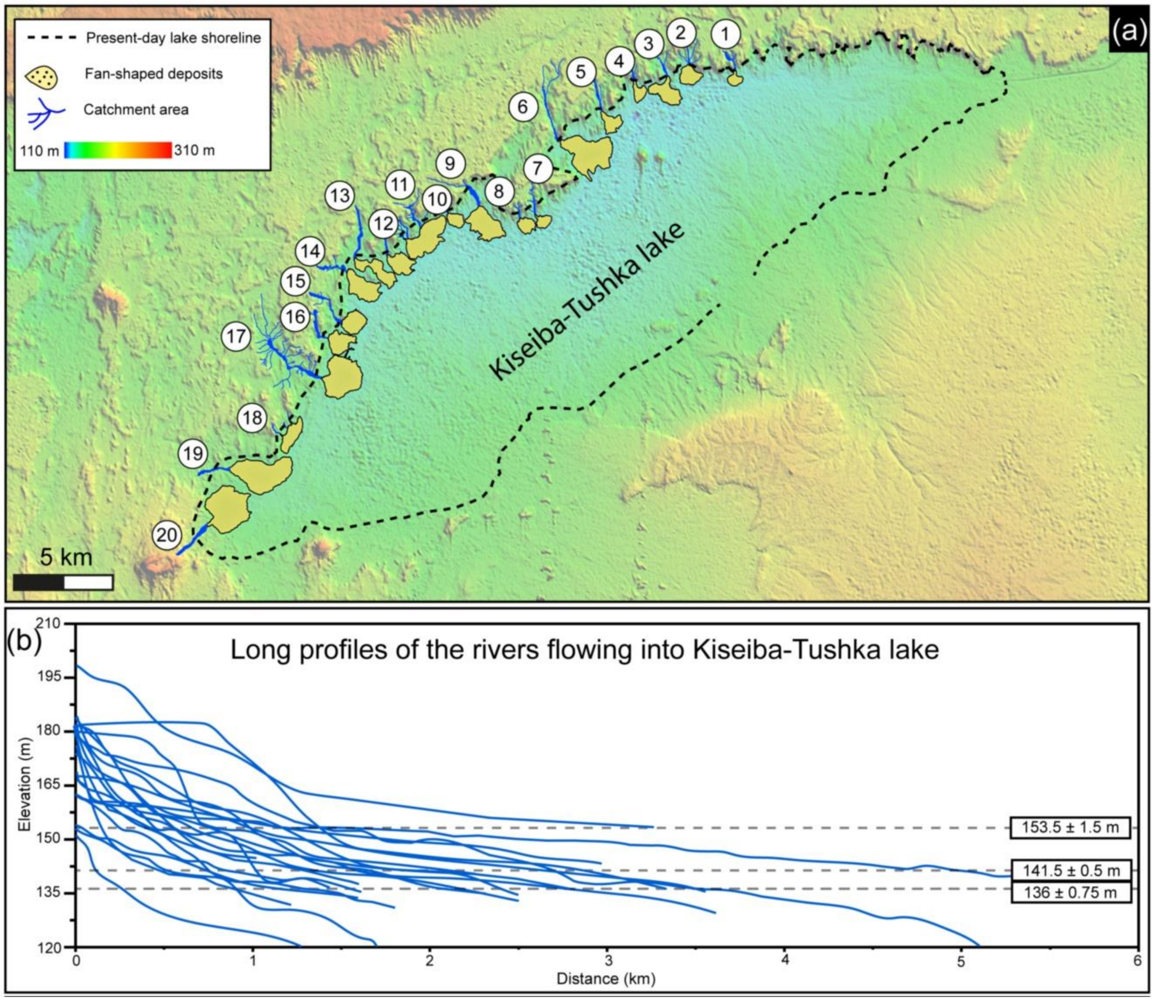

Figure S2. (A) ALOS PALSAR DEM shows the distribution of the deltaic features and their catchment areas. (b) Twenty long profiles of the main trunks of the rivers that drained into the lake. These long profiles indicate that the accumulation of the sediments took place at multiple lake levels.

Movie S1 and Movie S2. Animations showing the formation of deltaic features within lakes over the past ~30 years. These animations provide an idea of how much time is required for the continuous fluvial activity to form similar deltas accumulating within lakes; (1) Razzaza Lake, one of the modern Tushka lakes.
