# Supplementary figures and images for: Fluvial Depositional Systems of the African Humid Period: An Analog for an Early, Wet Mars in the Eastern Sahara
Source: J Geophys Res Planets. 2022 May 13;127(5):e2021JE007087. doi: 10.1029/2021JE007087 (PMC9285406; doi:10.1029/2021JE007087)

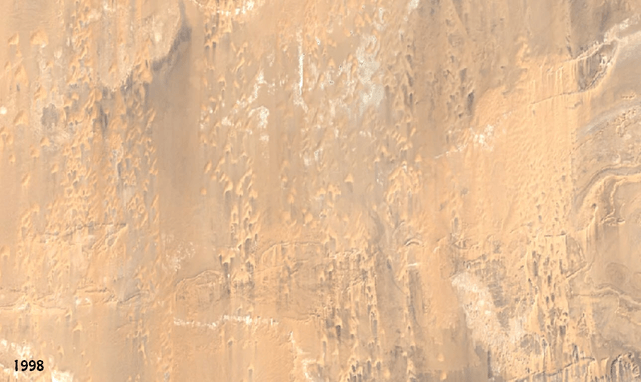

Supplement: Supplementary file 2 — Figure S1 [file JGRE-127-0-s003.gif]

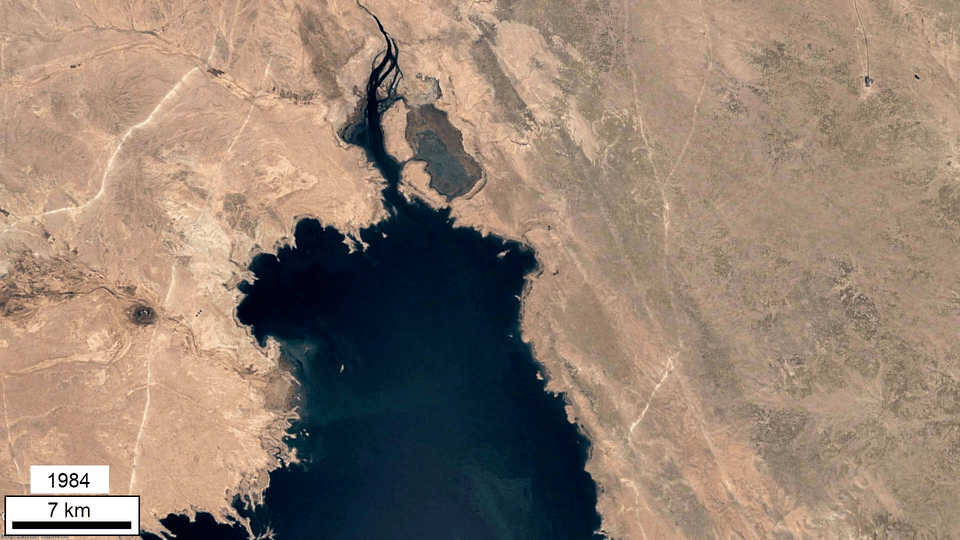

Supplement: Supplementary file 3 — Figure S2 [file JGRE-127-0-s002.gif]
